# Hierarchically porous activated carbon derived from *Lansium domesticum* peel *via* hydrothermal-H_3_PO_4_ activation for enhanced methylene blue removal: adsorption behavior, advanced modeling and mechanistic insights

**DOI:** 10.1039/d6ra02695h

**Published:** 2026-05-22

**Authors:** Tra Huong Do, Ngoc Phuong Ngan Nguyen, Xuan Truong Mai, Thi Hue Tran, Quoc Dung Nguyen, Truong Xuan Vuong

**Affiliations:** a Faculty of Chemistry, Thai Nguyen University of Education No. 20 Luong Ngoc Quyen Street Thai Nguyen City 24000 Vietnam; b Faculty of Natural Sciences and Technology, TNU-University of Science Tan Thinh Ward Thai Nguyen City 24000 Vietnam xuanvt@tnus.edu.vn

## Abstract

Dye-containing wastewater remains difficult to treat due to the persistence of synthetic dyes and the limitations of conventional adsorbents. In this study, a hierarchically porous activated carbon (ACLDP) was synthesized from *Lansium domesticum* peel *via* an integrated hydrothermal-H_3_PO_4_ activation strategy, yielding a mesopore-dominated structure enriched with oxygen-containing functional groups. Despite a moderate BET surface area (115.12 m^2^ g^−1^), the material exhibited a high adsorption capacity toward methylene blue (∼345.8 mg g^−1^), suggesting that adsorption performance is influenced not only by surface area but also by pore accessibility and surface chemistry. A statistically rigorous model discrimination approach based on the Akaike Information Criterion (AIC), which remains less commonly applied in dye adsorption studies, was employed to evaluate nonlinear isotherm and kinetic models. The Sips model provided the best description of equilibrium data (*R*^2^ = 0.9975, ΔAIC = 0), suggesting the relevance of surface heterogeneity in describing adsorption behavior. Kinetic analysis further indicated that no single model adequately captured the adsorption process, supporting a multi-step mechanism involving intraparticle diffusion and heterogeneous surface interactions. Mechanistic interpretation, supported by physicochemical characterization and adsorption behavior, suggests that electrostatic attraction, π–π interactions, hydrogen bonding, and pore diffusion collectively contribute to adsorption in a coupled and condition-dependent manner. These findings highlight that rational pore structure design and surface functionality can partially compensate for relatively low surface area, offering a viable strategy for converting agricultural residues into efficient carbon-based adsorbents. This study also provides a statistically supported framework for interpreting adsorption behavior, contributing to the development of sustainable materials aligned with circular economy principles.

## Abstract 2

Dye-containing wastewater remains difficult to treat due to the persistence of synthetic dyes and the limitations of conventional adsorbents.

## Graphical abstract

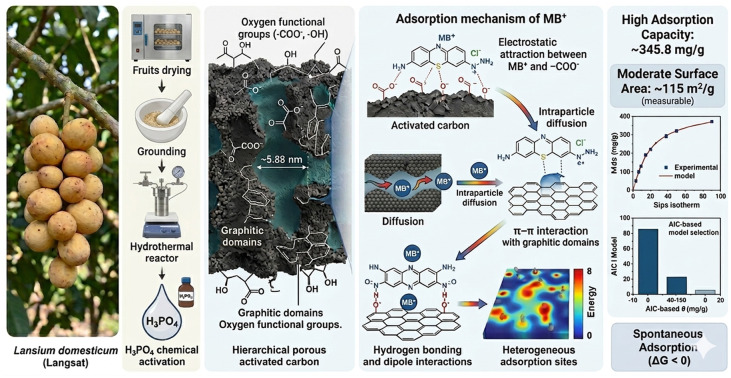

## 1. Introduction

Industrial growth in the textile and dyeing sectors continues to release large volumes of dye-laden wastewater into natural water systems. These effluents contain chemically stable organic compounds that resist biodegradation and persist over long periods, leading to cumulative ecological and human health risks.^1–3^ Among these contaminants, methylene blue (MB), a cationic thiazine dye, is frequently encountered due to its extensive industrial application and high chemical stability. Exposure to elevated MB concentrations has been linked to adverse health outcomes, including methemoglobinemia and neurological disorders.^4^ Current global production exceeds 7 × 10^5^ tons of synthetic dyes annually, with approximately 15–20% discharged into wastewater during processing stages.^5^ In regions undergoing rapid industrialization, such as Vietnam, wastewater treatment systems often develop more slowly than industrial output, allowing dye pollutants to enter aquatic environments with limited control.

Recent literature published between 2023 and 2026 continues to emphasize the persistence, toxicity, and structural complexity of synthetic dyes.^4,6,7^ These characteristics impose strict requirements on treatment technologies, which must combine efficiency with scalability and economic feasibility. A range of approaches has been explored, including membrane separation, advanced oxidation processes (AOPs), and biological treatments.^1,3^ Each route presents trade-offs. Membrane systems suffer from fouling and declining performance. AOPs demand high energy input and can generate secondary byproducts. Biological processes often fail to fully mineralize stable dye molecules. In contrast, adsorption offers a simpler operational framework and maintains high removal efficiency across varying concentrations.^2,8^ Yet practical deployment still faces two persistent challenges: the cost and regeneration limits of commercial adsorbents, and the difficulty of tailoring pore structure and surface chemistry to control adsorption kinetics and diffusion pathways.

To address these limitations, recent studies have increasingly explored biomass-derived porous carbons for dye-removal applications. Cao *et al.* reported that activated carbon derived from garden waste through deep-eutectic-solvent-assisted KOH activation exhibited enhanced methylene blue adsorption because of improved pore development and surface functionality.^9^ Similarly, Benmenine *et al.* prepared activated carbon from waste palm fiber and observed that both adsorption capacity and adsorption kinetics were strongly influenced by pore accessibility and oxygen-containing surface groups.^10^

Recent investigations have also emphasized that adsorption performance is governed not solely by BET surface area, but also by hierarchical pore architecture, diffusion behavior, and heterogeneous surface interactions. Gong *et al.* demonstrated that Fe_3_O_4_-N-modified banana-peel biochar achieved high methylene blue uptake through the combined contribution of electrostatic attraction, π–π interactions, and structural porosity.^11^ Related adsorption behavior was also discussed by Bouzgarrou *et al.*, who highlighted the importance of transport-controlled adsorption processes and surface heterogeneity in activated-carbon systems.^6^

Current review studies further indicate increasing interest in advanced biomass-derived adsorbents and mechanistic adsorption modeling. Adeoye *et al.* summarized recent progress in methylene blue adsorption technologies and emphasized the growing importance of mechanistic interpretation for improving adsorption prediction and process optimization.^4^ Onyango *et al.* additionally highlighted the emerging role of hydrochar-based materials and hierarchical porous structures in adsorption systems designed for aqueous dye removal.^5^

Despite these advances, the integration of hierarchical carbon engineering with statistically rigorous model discrimination approaches, particularly information-theoretic methods such as the Akaike Information Criterion (AIC), remains comparatively limited for region-specific biomass precursors such as *Lansium domesticum* peel.

Biomass-derived carbon materials provide an alternative route that aligns with sustainability goals. Lignocellulosic feedstocks are abundant and inexpensive, and they can be transformed into porous carbons with adjustable physicochemical properties.^9,10,12^ Compared with conventional activated carbons, these materials exhibit higher structural disorder, richer defect sites, and more diverse surface functionalities. Such features influence adsorption behavior at multiple scales. Hierarchical pore networks improve mass transport, while oxygen-containing groups contribute to surface polarity and electrostatic interactions.^13,14^

Despite these advantages, research efforts have largely centered on common precursors such as coconut shells, rice husk, and agricultural residues. Biomass specific to certain regions, especially those with distinct biochemical compositions, remains underexplored. This narrow focus limits broader understanding of how precursor chemistry shapes adsorption performance. In parallel, many studies still rely heavily on adsorption capacity as the primary evaluation metric. The interplay between pore hierarchy, surface chemistry, and transport mechanisms often receives less attention, even though it governs adsorption behavior at the molecular level.

Methylene blue (MB) was selected as the model adsorbate because it is one of the most widely employed representative cationic dyes in adsorption studies involving biomass-derived porous carbon materials. Its positively charged aromatic structure makes MB particularly suitable for evaluating electrostatic interactions, π–π interactions, pore accessibility, and diffusion-controlled adsorption behavior in heterogeneous carbon systems. In addition, the extensive availability of comparative adsorption data for MB enables more reliable benchmarking of adsorption capacity, adsorption kinetics, and statistical model discrimination across different adsorbent systems. Recent studies have similarly employed cationic dyes as representative probe molecules for evaluating adsorption behavior and mechanistic interactions in biomass-derived and polymer-modified adsorbents for wastewater treatment applications.^15–17^

*Lansium domesticum* peel represents one such overlooked precursor. This tropical fruit waste is abundantly available in Southeast Asia. Its composition includes lignocellulosic components enriched with phenolic and flavonoid compounds. During carbonization, these compounds can evolve into oxygen-containing surface groups such as hydroxyl and carboxyl functionalities. These chemical features are expected to influence adsorption through electrostatic attraction, hydrogen bonding, and π–π interactions with aromatic dye molecules.^18–20^

Although methylene blue adsorption using biomass-derived carbons has been extensively investigated, studies employing *Lansium domesticum* peel as a precursor remain limited. In particular, the combined use of hydrothermal-H_3_PO_4_ activation and information-theoretic adsorption model evaluation (AIC) has rarely been explored for this biomass system.

A second gap lies in the integration of material design and adsorption analysis. Conventional synthesis routes often employ single-step activation, restricting control over both pore development and surface functionality. Combining hydrothermal carbonization with chemical activation offers a more flexible approach. Hydrothermal treatment can pre-structure the carbon matrix while introducing oxygen functionalities. Subsequent phosphoric acid activation promotes pore formation through dehydration and crosslinking reactions, producing hierarchical porosity alongside reactive surface sites.^21–23^ Applications of this combined strategy to region-specific biomass, including *Lansium domesticum* peel, remain rare.

Limitations also appear in how adsorption data are interpreted. Many studies rely on correlation-based fitting, which does not adequately distinguish between competing models. The frequent use of the correlation coefficient (*R*^2^) can lead to ambiguous conclusions when multiple models provide similar fits. Information-theoretic approaches offer a different perspective. The Akaike Information Criterion (AIC) evaluates both goodness-of-fit and model complexity, reducing overfitting and improving model selection reliability.^24,25^ Despite these advantages, its use in adsorption studies is still limited.

More importantly, adsorption systems involving heterogeneous porous carbons frequently exhibit simultaneous contributions from surface-reaction kinetics, intraparticle diffusion, pore-filling effects, and heterogeneous-energy adsorption pathways. Under such conditions, different equilibrium and kinetic models may produce statistically comparable correlation coefficients despite representing fundamentally different physical mechanisms. This limitation can lead to mechanistic oversimplification and ambiguous interpretation of adsorption behavior in hierarchically porous biomass-derived carbons.

In this context, information-theoretic approaches such as the Akaike Information Criterion (AIC) provide more than a statistical goodness-of-fit comparison. By simultaneously accounting for fitting accuracy and model complexity, AIC enables discrimination between competing mechanistic interpretations while reducing overparameterization bias. The integration of AIC with error-function analysis therefore offers a more physically meaningful framework for identifying dominant adsorption pathways and transport behavior in heterogeneous porous carbon systems. Such an approach remains rarely implemented in adsorption studies involving biomass-derived activated carbons, despite its potential to improve mechanistic reliability and model interpretability.

Against this background, the present work adopts a dual strategy that addresses both material development and analytical rigor. This work combines hierarchical carbon structure engineering with information-theoretic adsorption analysis to resolve competing adsorption mechanisms and establish statistically robust relationships between pore architecture, surface chemistry, diffusion behavior, and adsorption performance in heterogeneous biomass-derived carbon systems. Activated carbon is produced from *Lansium domesticum* peel through a sequential process combining hydrothermal carbonization, phosphoric acid activation, and thermal treatment. The resulting material is characterized in terms of pore structure and surface chemistry. Adsorption behavior toward methylene blue is examined through equilibrium and kinetic analyses, with model selection supported by AIC alongside error functions such as RMSE and *χ*^2^. The present work primarily employs MB as a model cationic dye to investigate adsorption behavior in hierarchically porous carbon systems. Evaluation toward other dye classes, including anionic dyes and structurally distinct cationic dyes, remains important for future assessment of adsorption selectivity and broader applicability.

Rather than attributing adsorption performance solely to surface area, this study evaluates how pore hierarchy and surface functionalities collectively influence electrostatic interactions, π–π stacking, and intraparticle diffusion behavior. While these adsorption mechanisms have been extensively investigated in previous studies, the present work emphasizes a more rigorous analytical framework through the combined application of equilibrium/kinetic modeling, error-function analysis, and Akaike Information Criterion (AIC)-based model discrimination. This approach enables a statistically grounded interpretation of adsorption behavior and provides a more reliable basis for comparing competing adsorption models in biomass-derived carbon systems.

The findings provide further insight into how biomass-derived surface chemistry and pore structure influence adsorption behavior in carbon-based adsorbents. They also support the development of statistically informed approaches for evaluating dye adsorption performance in biomass-derived porous carbon systems.

## 2. Materials and methods

### 2.1. Materials

#### 2.1.1. Biomass precursor

Peels of *Lansium domesticum* were utilized as the lignocellulosic feedstock for the preparation of carbon-based materials. The raw biomass was obtained from nearby fresh fruit markets.

#### 2.1.2. Chemicals and reagents

All reagents employed in this work were of analytical grade and used as received without any additional purification steps.

• Activating agent: phosphoric acid (H_3_PO_4_, 40%) served as the chemical activator during the carbonization process.

• Neutralizing agent: a dilute sodium bicarbonate (NaHCO_3_) solution was employed to neutralize residual acidity after activation and to adjust the final material to near-neutral pH (approximately 7).

• Solvent: double-distilled water was consistently used for washing and solution preparation in order to prevent interference from extraneous ionic species that could affect the structural and surface characteristics of the synthesized materials.

• Ethanol was additionally applied during the washing step to remove residual organic impurities and improve the cleanliness of the final product.

### 2.2. Preparation of activated carbon

Activated carbon was synthesized from *Lansium domesticum* peel through an integrated process comprising hydrothermal carbonization, chemical activation, and subsequent thermal treatment under an inert atmosphere.

#### 2.2.1. Step 1: raw material preparation

The collected *Lansium domesticum* peels were thoroughly rinsed several times with distilled water to remove surface-adhered impurities and residual organic matter. The cleaned biomass was then air-dried or oven-dried at 80 °C for 24 h until complete moisture removal was achieved. The dried material was subsequently crushed and sieved to obtain particles in the size range of 2–5 mm. The prepared precursor was denoted as LDP (*Lansium domesticum* peel).

#### 2.2.2. Step 2: hydrothermal carbonization

The prepared LDP was dispersed in distilled water at a solid-to-liquid ratio of 1 : 10 (g mL^−1^) and transferred into a sealed stainless-steel autoclave. The hydrothermal treatment was conducted at 200 °C for 6 h under autogenous pressure. After the reaction, the system was allowed to cool naturally to room temperature. The resulting solid product (hydrochar) was collected, thoroughly washed with distilled water to remove soluble by-products, and dried at 105 °C until a constant weight was obtained.

#### 2.2.3. Step 3: chemical activation with H_3_PO_4_

A 40% phosphoric acid (H_3_PO_4_) solution was prepared and used as the activating agent. The dried hydrochar was impregnated with the H_3_PO_4_ solution at a mass ratio of H_3_PO_4_ to hydrochar of 1 : 3. The mixture was maintained under mild stirring at room temperature for 24 h to ensure sufficient penetration and interaction of the activating agent with the carbon matrix. After impregnation, the sample was first treated with a dilute NaHCO_3_ solution to neutralize residual acidity, followed by extensive washing with distilled water until the filtrate reached near-neutral pH and was free of residual phosphate species. The sample was then filtered and dried at 105 °C to constant weight.

#### 2.2.4. Step 4: carbonization (pyrolysis)

The impregnated sample was placed in a ceramic crucible and subjected to thermal treatment in a tubular furnace under a continuous nitrogen atmosphere. The temperature was increased to 500 °C at a heating rate of 5 °C min^−1^ and maintained for 2 h. After carbonization, the furnace was allowed to cool naturally to room temperature under nitrogen flow. The resulting activated carbon was collected and stored in an airtight desiccator to prevent moisture adsorption and contamination. The final product was designated as ACLDP (activated carbon derived from *Lansium domesticum* peel).

**Fig. 1:**
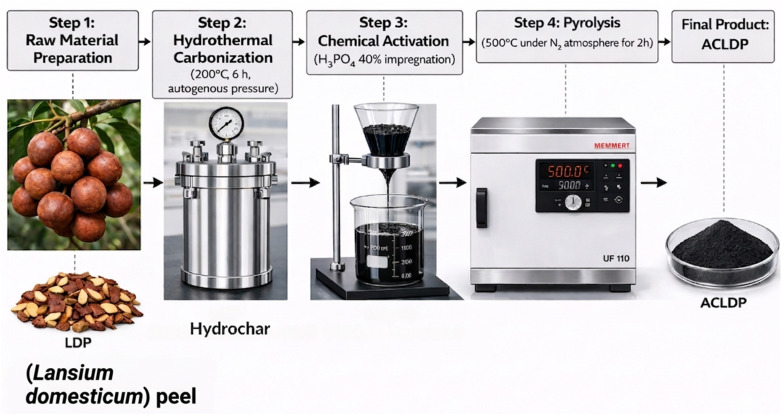
Schematic diagram for the synthesis of ACLDP.

The overall preparation procedure is schematically illustrated in Fig. 1.

### 2.3. Characterization of materials

#### 2.3.1. Textural properties (BET analysis)

The specific surface area and pore structure of ACLDP were determined by N_2_ adsorption–desorption measurements at 77 K using a TriStar II 3020 analyzer (Micromeritics, USA). Prior to analysis, samples were degassed under vacuum to remove moisture and physisorbed species. The specific surface area was calculated using the Brunauer–Emmett–Teller (BET) method, while the total pore volume was estimated at a relative pressure of *p*/*p*_0_ ≈ 0.99. The pore size distribution was derived from the desorption branch using the BJH model. Isotherms were classified according to IUPAC recommendations.

#### 2.3.2. Crystalline structure (XRD)

The crystalline phases of ACLDP were analyzed using X-ray diffraction (XRD, Equinox 5000, Thermo Scientific, France) with Cu Kα radiation (*λ* = 1.5406 Å). Diffraction patterns were recorded over a 2*θ* range of 10–80° with a step size of 0.02° and a scanning rate of 2° min^−1^. Phase identification was performed using the PDF database, and interplanar spacing (*d*) was calculated based on Bragg's law. Peak features were evaluated to assess structural ordering and phase stability.

#### 2.3.3. Morphology and elemental analysis (SEM-EDS, TEM)

Surface morphology of LDP and ACLDP before and after adsorption was observed using scanning electron microscopy (SEM, JSM-6510LV, JEOL, Japan). Elemental composition and distribution were determined by energy-dispersive X-ray spectroscopy (EDS) coupled with SEM.

#### 2.3.4. Surface functional groups (FTIR)

Functional groups of ACLDP before and after adsorption were identified by Fourier transform infrared spectroscopy (FTIR, Nicolet Nexus 670, Thermo Scientific, USA) over the range of 4000–400 cm^−1^. The spectra were used to evaluate changes in surface chemistry and possible interactions between functional groups and adsorbate molecules.

#### 2.3.5. Raman spectroscopy

The structural characteristics and degree of graphitization of ACLDP were analyzed using a Micro-Raman spectrometer (LABRAM-1B, Jobin-Yvon, France). The intensity ratio of the D and G bands (*I*_D_/*I*_G_) was used to assess the defect density and structural ordering of the carbon framework.

#### 2.3.6. Thermogravimetric analysis (TGA)

Thermal stability of ACLDP was evaluated by thermogravimetric analysis (TGA) using a NETZSCH TG 209 F3 Tarsus instrument (Germany). The weight loss profile was recorded as a function of temperature to assess decomposition behavior and thermal resistance.

#### 2.3.7. Determination of pH_pzc_

The point of zero charge (pH_pzc_) of ACLDP was determined using the pH drift method. A 0.01 M NaCl solution was employed as the background electrolyte to maintain constant ionic strength. The initial pH (pH_0_) of a series of 50 mL NaCl solutions was adjusted within the range of 2–11 using 0.1 M HCl or 0.1 M NaOH. Subsequently, 0.05 g of ACLDP was added to each solution, and the suspensions were agitated at 150 rpm for 24 h at 25 ± 1 °C to reach equilibrium.

After equilibration, the final pH (pH_f_) was recorded, and the pH variation (ΔpH) was calculated as:

1 ΔpH = pH_f_ − pH_0_

The pH_pzc_ was identified as the point at which ΔpH = 0 from the plot of ΔpH *versus* pH_0_. This parameter was used to assess the surface charge behavior of ACLDP and to elucidate electrostatic interactions between the adsorbent and methylene blue under different pH conditions.

#### 2.3.8. Zeta potential

Zeta potential measurements were carried out using a Zetasizer Nano ZS analyzer (Malvern Instruments Ltd, UK) operating on the basis of electrophoretic light scattering (ELS), coupled with a disposable folded capillary zeta cell. Approximately 0.01 g of ACLDP was dispersed in 50 mL of deionized water. Prior to analysis, the suspension was ultrasonicated for 15 min to improve particle dispersion homogeneity. The suspension pH was adjusted to the desired values using dilute HCl or NaOH solutions, and all measurements were performed at 25 ± 0.1 °C.

Electrophoretic mobility data were converted into zeta potential values through the Smoluchowski approximation integrated within the instrument software (Zetasizer Software Ver. 7.11). Instrument settings included a dispersant refractive index of 1.330, viscosity of 0.8872 cP, and dielectric constant of 78.5. Each sample underwent triplicate analysis, with every measurement consisting of at least 10 instrument runs. Reported values correspond to the mean ± standard deviation.

### 2.4. Batch adsorption experiments

Batch adsorption experiments were carried out to assess the effects of key operational parameters on methylene blue (MB) removal by ACLDP. All tests were performed in 100 mL conical flasks. Upon completion, the suspensions were centrifuged at 4000 rpm for 15 min to separate the solid phase, and the residual MB concentration was quantified.

All experiments were conducted in triplicate, and the reported values represent the mean results. The removal efficiency (% *H*) and adsorption capacity at time *t* (*q*_*t*_, mg g^−1^) were calculated using the following equations: The removal efficiency (% *H*) and adsorption capacity at time *t* (*q*_*t*_, mg g^−1^) were calculated as follows:2
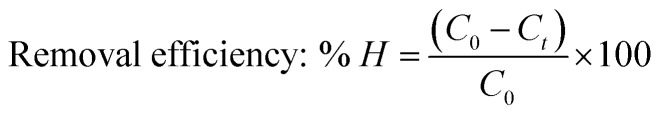
3
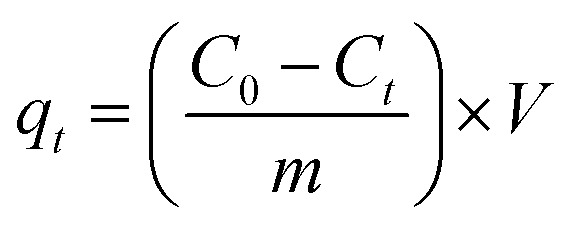
where *C*_0_ and *C*_*t*_ (mg L^−1^) denote the initial and time-dependent MB concentrations, respectively, *V* (L) is the solution volume, and *m* (g) is the mass of adsorbent.

MB concentration was determined by UV-Vis spectrophotometry at a maximum wavelength of 664 nm. Calibration was performed within the range of 1–10 mg L^−1^, yielding a strong linear correlation (*R*^2^ > 0.998), which ensured reliable quantification. Detailed calibration data are provided in the SI (Fig. S1). For samples with MB concentrations exceeding the linear calibration range (0–10 mg L^−1^), appropriate dilution with distilled water was performed prior to UV-Vis measurement to ensure that absorbance values remained within the Beer–Lambert linear region.

#### 2.4.1. Effect of pH

The influence of solution pH on MB adsorption was examined over the range of 3–10. In each experiment, 0.03 g of ACLDP was introduced into 25 mL of MB solution (initial concentration ≈ 50 mg L^−1^). The pH was adjusted using 0.1 M HCl or 0.1 M NaOH prior to adsorption. The suspensions were agitated at 300 rpm for 90 min at 25 ± 1 °C.

After the adsorption process, the mixtures were centrifuged at 4000 rpm for 15 min to separate the solid phase, and the residual MB concentration in the supernatant was subsequently determined.

#### 2.4.2. Effect of contact time

Adsorption kinetics were evaluated by introducing 0.03 g of ACLDP into 25 mL of MB solutions with nominal initial concentrations of 58, 87, and 117 mg L^−1^ at pH 7. The experimentally measured concentrations after solution preparation were 58.51, 87.76, and 117.01 mg L^−1^, respectively. The mixtures were agitated at 300 rpm at 25 ± 1 °C over predetermined time intervals (10–150 min).

At each selected time, samples were withdrawn and centrifuged at 4000 rpm to separate the solid phase. The residual MB concentration in the supernatant was then determined.

#### 2.4.3. Effect of initial concentration

The effect of initial MB concentration was investigated over the range of 50–500 mg L^−1^. In each experiment, 0.03 g of ACLDP was added to 25 mL of MB solution at pH 7. The suspensions were agitated at 300 rpm for 90 min at 25 ± 1 °C.

Following adsorption, the mixtures were centrifuged at 4000 rpm for 15 min to separate the solid phase, and the residual MB concentration in the supernatant was subsequently determined.

#### 2.4.4. Effect of adsorbent dosage

ACLDP with varying dosages (0.01–0.05 g) was introduced into 100 mL conical flasks. Subsequently, 25 mL of MB solution with an accurately determined initial concentration of 50 mg L^−1^ was added to each flask, and the solution pH was adjusted and maintained at 7.

The adsorption process was carried out under agitation at 300 rpm for 90 min at room temperature (25 ± 1 °C). Upon completion, the samples were centrifuged at 4000 rpm for 15 min to separate the solid phase, and the residual MB concentration in the supernatant was determined.

#### 2.4.5. Effect of temperature

The influence of temperature on MB adsorption was investigated at 303, 313, and 323 K. In each experiment, 0.03 g of ACLDP was added to 25 mL of MB solution (initial concentration ≈ 100 mg L^−1^) at pH 7, adjusted using 0.1 M NaOH or 0.1 M HNO_3_. The adsorption process was conducted under continuous stirring (300 rpm) on a temperature-controlled magnetic stirrer for 30–120 min. After adsorption, the suspensions were centrifuged to separate the solid phase, and the residual MB concentration in the supernatant was determined. The obtained data were used to evaluate the thermodynamic characteristics of the adsorption process.

### 2.5. Adsorption modeling

Adsorption data were analyzed using kinetic, isotherm, and thermodynamic models to elucidate the mechanism of methylene blue (MB) uptake onto ACLDP. Model parameters were estimated by nonlinear regression through minimization of the sum of squared errors (SSE), ensuring accurate fitting to experimental data. Model performance was evaluated using multiple statistical indicators, including the coefficient of determination (*R*^2^), chi-square (*χ*^2^), root mean square error (RMSE), and Akaike information criterion (AIC). While *R*^2^ reflects the correlation between predicted and experimental values, *χ*^2^ and RMSE quantify residual deviations, and AIC assesses model quality by accounting for both goodness-of-fit and model complexity. This multi-criteria approach enables robust and unbiased model comparison, facilitating reliable identification of the most appropriate model.

#### 2.5.1. Kinetic models

Adsorption kinetics were investigated using a combination of surface-reaction, diffusion-controlled, and heterogeneous-site kinetic models in order to resolve the dominant rate-governing mechanisms during methylene blue uptake. The selected kinetic frameworks encompass adsorption systems exhibiting different energetic distributions, interfacial transport characteristics, and diffusion pathways.

Surface-reaction kinetics were first evaluated using pseudo-first-order (PFO), pseudo-second-order (PSO), and Elovich models. The nonlinear PFO model was expressed as:

4 *q*_*t*_ = *q*_e_[1 − exp(−*k*_1_*t*)]

where *q*_*t*_ and *q*_e_ (mg g^−1^) denote the adsorption capacities at time *t* and equilibrium, respectively, and *k*_1_ (min^−1^) is the pseudo-first-order rate constant.

The PSO model was represented by:5
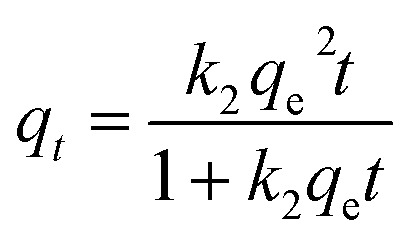
where *k*_2_ (mg g^−1^ min^−1^) is the pseudo-second-order rate constant. This model is frequently associated with adsorption systems involving surface-site occupation and stronger adsorbate–surface interactions.

The Elovich kinetic equation was written as:6
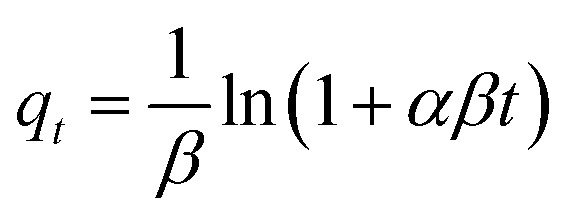
where *α* (mg g^−1^ min^−1^) represents the initial adsorption rate and *β* (g mg^−1^) reflects energetic heterogeneity and activation-related surface effects.

Diffusion-controlled transport behavior was additionally examined using Weber–Morris intraparticle diffusion and liquid-film diffusion models. The Weber–Morris equation was expressed as:

7 *q*_*t*_ = *k*_id_*t*^0.5^ + *C*

where *k*_id_ (mg g^−1^ min^−0.5^) is the intraparticle diffusion rate constant and *C* corresponds to the boundary-layer contribution. Multi-linear diffusion profiles were interpreted as evidence of sequential adsorption stages involving external diffusion, pore diffusion, and equilibrium adsorption.

External mass-transfer resistance was further evaluated using the liquid-film diffusion model:

8 ln(1 − *F*) = −*k*_f_*t*

where *k*_f_ (min^−1^) is the liquid-film diffusion constant and *F* is the fractional attainment of equilibrium defined as: 9
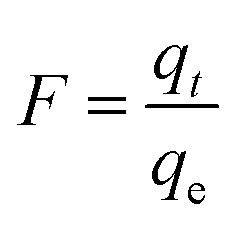


To account for adsorption systems exhibiting time-dependent kinetic heterogeneity and complex adsorption pathways, the Avrami kinetic model was also applied:

10 *q*_*t*_ = *q*_e_[1 − exp(−*k*_AV_*t*^*n*^)]

where *k*_AV_ is the Avrami kinetic constant and *n* is the Avrami exponent associated with adsorption-pathway complexity and temporal evolution of adsorption rates.

All kinetic parameters, including *k*_1_, *k*_2_, *k*_id_, *k*_f_, *k*_AV_, *n*, *α*, *β*, and the calculated equilibrium adsorption capacity (*q*_e,cal_), were estimated using nonlinear regression implemented through the Levenberg–Marquardt optimization algorithm in the minpack.lm package in R.

Model discrimination was performed using multiple statistical and information-theoretic criteria, including the coefficient of determination (*R*^2^), chi-square (*χ*^2^), root mean square error (RMSE), Akaike information criterion (AIC), and sum of squared errors (SSE). The simultaneous use of error-based and information-theoretic metrics reduces overfitting bias and improves discrimination among competing kinetic mechanisms.

11 SSE = ∑(*q*_e,exp_ − *q*_e,cal_)^2^

Higher *R*^2^ values together with lower *χ*^2^, RMSE, AIC, and SSE values indicate improved agreement between model predictions and experimental observations.

The apparent activation energy (*E*_a_) was further evaluated using the Arrhenius relationship:12
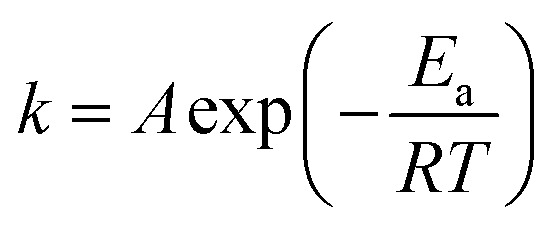
which can be linearized as:13
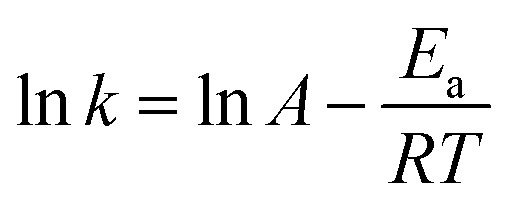
where *A* is the Arrhenius pre-exponential factor, *R* is the universal gas constant (8.314 J mol^−1^ K^−1^), and *T* is the absolute temperature (K). The magnitude of *E*_a_ was interpreted in relation to diffusion resistance and adsorbate–surface interaction strength. Activation energies below approximately 40 kJ mol^−1^ are commonly associated with physisorption-dominated systems, whereas larger values may indicate stronger surface interactions or chemisorption contributions.

#### 2.5.2. Isotherm models

Equilibrium adsorption behavior was systematically analyzed using Langmuir, Freundlich, Temkin, Sips, Toth, and Dubinin–Radushkevich (D–R) isotherm models. The combined use of these mechanistically distinct models allowed evaluation of monolayer, multilayer, and heterogeneous adsorption processes, as well as adsorption systems with non-uniform energy distributions, thereby providing deeper insight into the dominant adsorption pathways.

Monolayer adsorption behavior was first examined using the Langmuir isotherm model:14
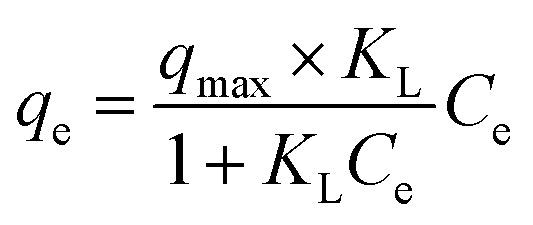
where *q*_e_ (mg g^−1^) is the equilibrium adsorption capacity, *q*_max_ (mg g^−1^) is the maximum monolayer adsorption capacity, *b* (L mg^−1^) is the Langmuir affinity constant, and *C*_e_ (mg L^−1^) is the equilibrium adsorbate concentration.

Adsorption favorability was assessed using the dimensionless separation factor:15
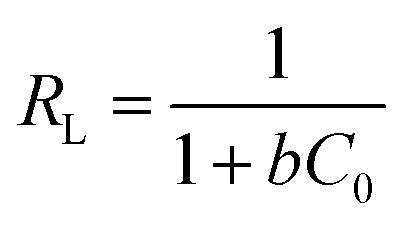
where *C*_0_ is the initial adsorbate concentration. Values of 0 < *R*_L_ < 1 indicate favorable adsorption.

Surface heterogeneity and multilayer adsorption behavior were further analyzed using Freundlich, Sips, Toth, Elovich, and Halsey isotherm frameworks.

The Freundlich model was represented as:

16 *q*_e_ = *K*_F_*C*_e_^1/*n*^

where *K*_F_ is the Freundlich adsorption constant and *n* is the heterogeneity parameter associated with adsorption intensity.

The Temkin model was expressed as:17
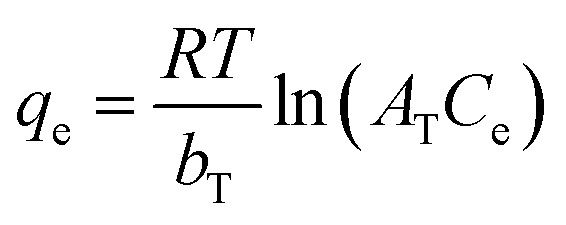
where *A*_T_ is the Temkin equilibrium binding constant and *b*_T_ is associated with adsorption heat distribution across the adsorbent surface.

The Sips model was represented by:18
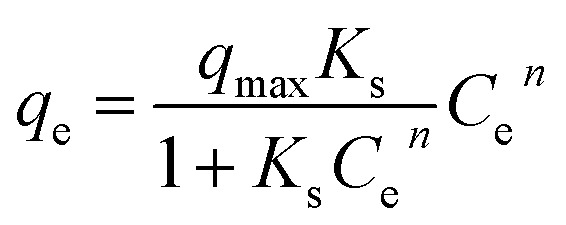
where *K*_s_ is the Sips equilibrium constant and ns is the heterogeneity parameter. The Sips equation combines Langmuir-type saturation behavior with Freundlich-type heterogeneity.

The Toth model was expressed as:19
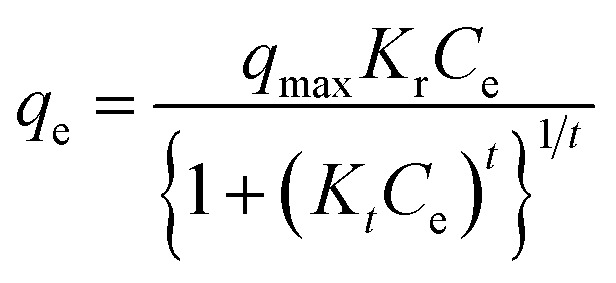
where *K*_T_ is the Toth equilibrium constant and *t* represents deviation from ideal Langmuir behavior.

Adsorption-energy distribution and pore-filling characteristics were additionally investigated using the Dubinin–Radushkevich (D–R) model:

20 *q*_e_ = *q*_m_ exp(−*βε*^2^)

where *q*_m_ is the theoretical saturation capacity and *β* is the adsorption-energy coefficient.

The Polanyi potential was calculated as:21
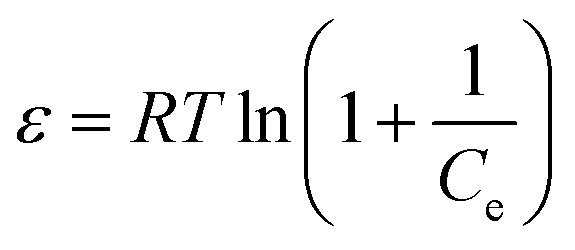


The mean adsorption energy (*E*) was estimated using:22
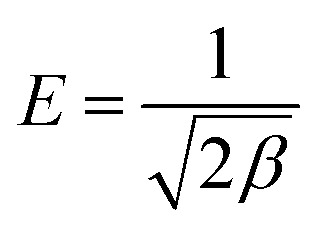
The magnitude of *E* was used as a qualitative indicator of the dominant adsorption interaction, including possible contributions from physisorption, ion exchange, and stronger surface interactions.

The Elovich isotherm equation was written as:23
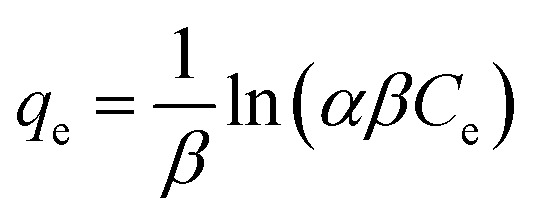
where *α* and *β* are constants associated with adsorption capacity and energetic heterogeneity.

Multilayer adsorption over heterogeneous porous surfaces was additionally assessed using the Halsey model:24
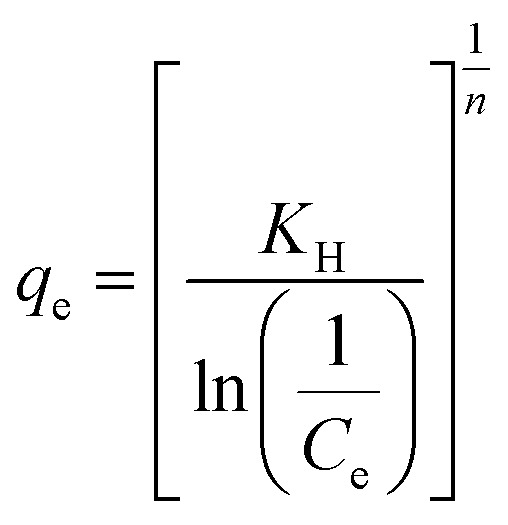
where *K*_H_ is the Halsey adsorption constant and *n* is the heterogeneity parameter.

All nonlinear fitting procedures were implemented in R using the minpack.lm package based on the Levenberg–Marquardt optimization algorithm. This approach minimizes residual errors without introducing mathematical distortions associated with linearization procedures.

Model selection and fitting quality were evaluated using the coefficient of determination (*R*^2^), chi-square (*χ*^2^), root mean square error (RMSE), Akaike information criterion (AIC), and sum of squared errors (SSE). The combined use of error-based and information-theoretic metrics enables statistically robust discrimination among competing equilibrium models.

25 *R*^2^ = 1 − [Σ(*q*_e,exp_ − *q*_e,cal_)^2^/Σ(*q*_e,exp_ − *q̄*_e,exp_)^2^]

26 *χ*^2^ = Σ[(*q*_e,exp_ − *q*_e,cal_)^2^/*q*_e,cal_]

27 RMSE = √[(1/*N*)Σ(*q*_e,exp_ − *q*_e,cal_)^2^]

28 AIC = *N* ln(RSS/*N*) + 2*p*

29 SSE = Σ(*q*_e,exp_ − *q*_e,cal_)^2^

where *q*_e,exp_ and *q*_e,cal_ are the experimental and calculated adsorption capacities, respectively, *N* is the number of observations, RSS is the residual sum of squares, and *p* is the number of fitted parameters.

For AIC calculation, the number of adjustable parameters (*k*) was defined as follows: PFO (2), PSO (2), Elovich kinetic (2), Weber–Morris (2), liquid-film diffusion (1), Avrami (3); Langmuir (2), Freundlich (2), Temkin (2), Dubinin–Radushkevich (2), Sips (3), Toth (3), Elovich isotherm (2), and Halsey (2).

#### 2.5.3. Thermodynamic analysis

Thermodynamic parameters were evaluated using the Van't Hoff approach in order to assess adsorption spontaneity, heat effects, and interfacial disorder during methylene blue adsorption.

The apparent distribution coefficient was calculated as:

30 *K*_D_ = *q*_e_/*C*_e_

where *K*_D_ was employed as an approximation of the thermodynamic equilibrium constant under dilute solution conditions.

The standard Gibbs free energy change was determined using:

31 Δ*G*° = −*RT* ln *K*_D_

where *R* is the universal gas constant (8.314 × 10^−3^ kJ mol^−1^ K^−1^) and *T* is the absolute temperature (K).

The Van't Hoff equation was expressed as:32
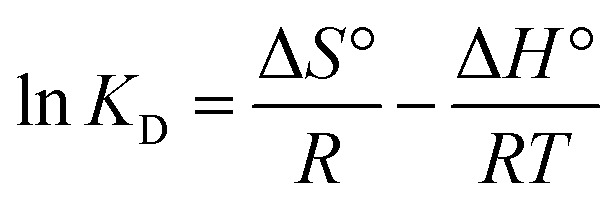
where Δ*H*° (kJ mol^−1^) and Δ*S*° (J mol^−1^ K^−1^) represent the standard enthalpy and entropy changes, respectively.

The values of Δ*H*° and Δ*S*° were determined from the slope and intercept of the linear plot of ln *K*_D_*versus* 1/*T*. Negative values of Δ*G*° indicate spontaneous adsorption, whereas positive and negative values of Δ*H*° correspond to endothermic and exothermic adsorption processes, respectively.

### 2.6. Regeneration study

#### 2.6.1. Desorption procedure

After the adsorption step, MB-loaded ACLDP was subjected to solvent-based regeneration. The exhausted adsorbent was treated with 70% (v/v) ethanol under mild stirring to weaken and remove the interactions between adsorbed MB molecules and the adsorbent surface. Subsequently, the material was thoroughly rinsed with distilled water to eliminate residual solvent and desorbed species, and then dried to constant mass prior to reuse.

#### 2.6.2. Reusability assessment

The reusability of ACLDP was investigated over five successive adsorption–desorption cycles under identical experimental conditions. In each cycle, 0.03 g of adsorbent was added to 25 mL of MB solution (initial concentration range: 50–500 mg L^−1^) at pH 7. The adsorption process was conducted on a shaker at 300 rpm for 90 min at 25 ± 1 °C. After adsorption, the suspensions were centrifuged at 4000 rpm for 15 min, and the residual MB concentration in the supernatant was determined.

For regeneration, the spent adsorbent was treated with 70% ethanol under stirring for 60 min, followed by thorough washing with distilled water and drying at 80 °C for 12 h before the next cycle.

The structural integrity of the regenerated material was evaluated by XRD analysis after the third cycle and compared with that of the fresh sample. The regeneration efficiency was determined based on the retention of removal performance relative to the initial cycle.

### 2.7. Statistical analysis

All experiments were performed in at least triplicate, and the data are reported as mean ± standard deviation (SD). Statistical analyses were carried out using R software (version 4.5.2, R Foundation for Statistical Computing, Vienna, Austria). Data visualization was conducted using OriginPro 2019 and R, with RStudio (Posit, Boston, MA, USA) employed as the integrated development environment.

## 3. Results and discussion

### 3.1. Textural & structural properties of materials

#### 3.1.1. The X-ray diffraction analysis

**Fig. 2:**
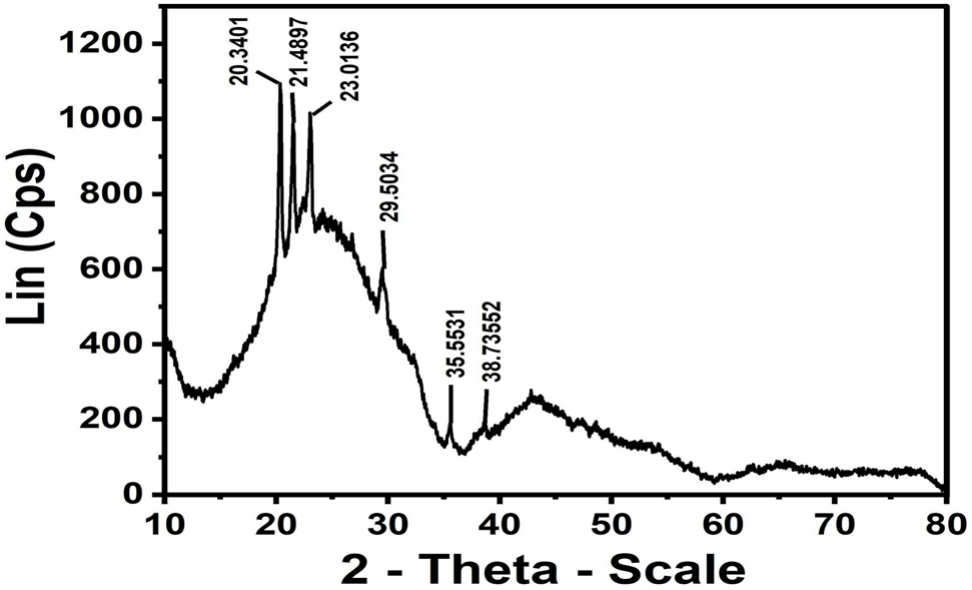
XRD pattern of ACLDP.

The X-ray diffraction (XRD) pattern of ACLDP (Fig. 2) exhibits features typical of a poorly ordered porous carbon material. A broad diffraction band appears in the 2*θ* range of approximately 20–25°, with discernible maxima at 20.34°, 21.49°, and 23.01°, corresponding to interplanar spacings (*d*) of about 4.38, 4.11, and 3.89 Å, respectively. This diffuse band is associated with the (002) reflection of graphitic carbon layers lacking regular stacking, reflecting a turbostratic arrangement in which graphene-like sheets are misaligned and randomly oriented. Such a structure is consistent with limited crystallinity and a dominant amorphous carbon phase.

The broadening and relatively low intensity of the (002) feature suggest limited coherent domain size and a considerable degree of structural disorder. These characteristics are commonly observed in biomass-derived activated carbons subjected to chemical activation, where partial disruption of graphitic ordering occurs. Additional weak diffraction features are observed at approximately 29.50°, 35.55°, and 38.74°. These signals may be attributed to trace inorganic phases or residual mineral species originating from the precursor or introduced during the activation process. Their weak intensity and poor definition indicate a minor contribution to the overall structure.

Taken together, the diffraction profile is consistent with a predominantly amorphous carbon framework with low structural order and significant disorder. Such structural features are typically associated with porous carbon materials and are often discussed in relation to adsorption applications involving organic molecules such as methylene blue (MB).^26^

#### 3.1.2. Raman and FT_IR

##### 3.1.2.1 FT-IR

**Fig. 3:**
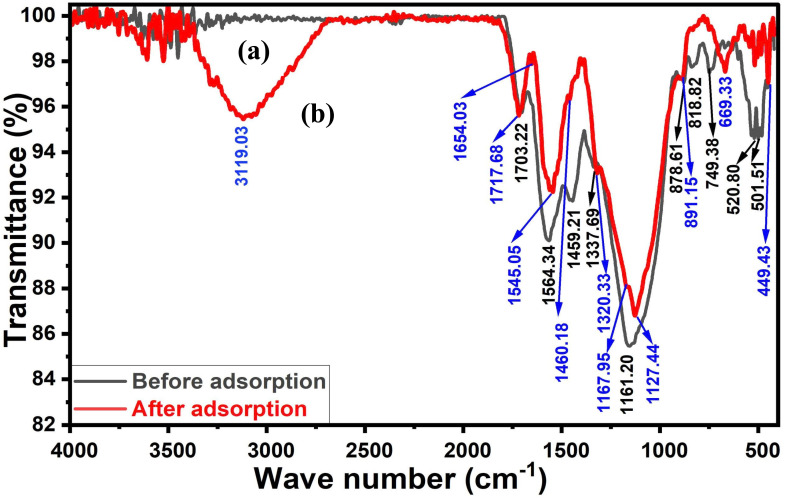
FT-IR spectra of ACLDP before (a) and after MB adsorption (b).

FT-IR spectroscopy was employed to examine the surface functional groups of ACLDP and their evolution following interaction with methylene blue (MB). The spectrum of the pristine material (Fig. 3a) exhibits a band at 1703 cm^−1^, assigned to the stretching vibration of carbonyl-containing groups (–COO^−^/C=O). A band at 1564 cm^−1^ is associated with C=C vibrations within aromatic domains of the carbon framework, whereas features in the 1337–1161 cm^−1^ region correspond to C–O stretching vibrations of oxygen-containing functionalities such as phenolic and ether groups.^20^

After MB adsorption (Fig. 3b), several spectral modifications become apparent. A broad absorption band appears at approximately 3119 cm^−1^, located within the characteristic region commonly associated with O–H stretching vibrations of hydroxyl groups and adsorbed moisture, with possible contributions from aromatic C–H vibrations and weak N–H-related interactions originating from adsorbed MB molecules. Changes in this spectral region suggest participation of surface functional groups during the adsorption process.

The carbonyl-related band shifted slightly from 1703 to 1717 cm^−1^ after adsorption, reflecting modification of the local chemical environment of oxygen-containing surface groups and possible involvement in adsorption-related interactions. Variations observed in the 1654–1545 cm^−1^ region and near 1320 cm^−1^ coincide with vibrational features commonly associated with aromatic C=C and C–N groups of MB, supporting the presence of adsorbed dye species on the ACLDP surface.

The observed spectral modifications are chemically consistent with adsorption-related interactions occurring at the ACLDP surface. π–π interactions between aromatic domains of the carbon framework and MB molecules remain chemically plausible because both structures contain conjugated aromatic systems. Oxygen-containing surface functionalities may additionally participate through hydrogen-bond-related interactions and electrostatic attraction with cationic MB species (MB^+^).^27^

While FT-IR alone does not allow definitive distinction among these interaction pathways, the observed spectral changes support the coexistence of multiple adsorption mechanisms in the porous carbon system.^27^ The summary of FT-IR band assignments of ACLDP before and after MB adsorption is presented in Table 1.

**Table 1:** FT-IR band assignments of ACLDP before and after MB adsorption^28^

Before adsorption (cm^−1^)	After adsorption (cm^−1^)	Functional group	Vibration mode	Interpretation
	3119	O–H/aromatic C–H/possible weak N–H contribution	Stretching	Presence of adsorbed MB and surface hydrogen-bond interactions
1703	1717	C=O	Stretching	Participation of carbonyl groups in hydrogen bonding or electrostatic interactions
1564	1654–1545	Aromatic C=C	Stretching	Possible π–π interaction between MB aromatic rings and carbon framework
1337	1320	C–O/C–N	Stretching	Interaction between surface functional groups and MB molecules
1161	1167–1127	C–O–C/C–N	Stretching	Changes associated with hydrogen-bond-related interactions
878–818	891–834	Aromatic C–H	Bending	Aromatic ring structure associated with MB molecules

##### 3.1.2.2 Raman

**Fig. 4:**
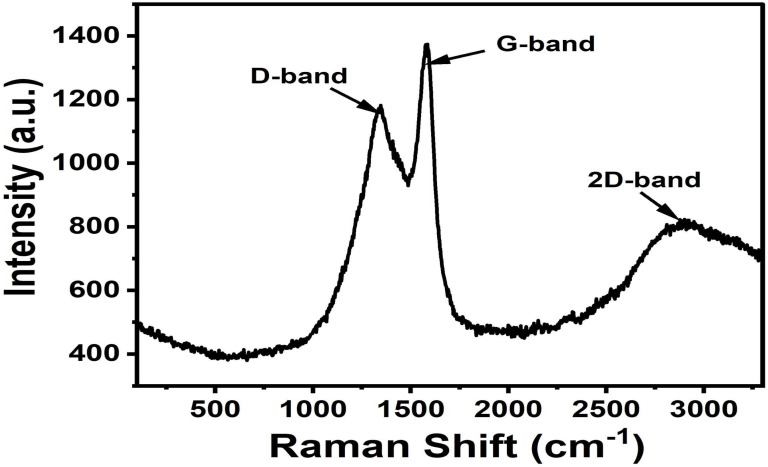
Raman spectrum of ACLDP.

The Raman spectrum of ACLDP (Fig. 4) is characterized by the typical signatures of sp^2^-hybridized carbon, with two prominent bands located at ∼1350 cm^−1^ (D band) and ∼1580 cm^−1^ (G band), along with a broad second-order feature in the 2700–3000 cm^−1^ region. The G band originates from the in-plane stretching vibration of C=C bonds (E_2g_ mode), which is associated with ordered graphitic domains. In contrast, the D band is defect-activated and arises from disruptions in the sp^2^ carbon lattice, including edge defects, vacancies, and the finite size of graphitic crystallites.^29^

The intensity ratio (*I*_D_/*I*_G_) is approximately 0.9, suggesting a relatively high level of structural disorder and limited graphitic domain size. According to the Tuinstra–Koenig relation, this ratio is inversely related to the in-plane crystallite size (*L*_a_), which is consistent with the presence of small graphitic domains embedded within a disordered carbon matrix.^30^ Such features are commonly observed in biomass-derived activated carbons subjected to chemical activation, where the development of porosity is accompanied by fragmentation and distortion of graphitic layers.^31^

The 2D band appears broad and of lower intensity compared to the G band, lacking the sharp and symmetric profile characteristic of single-layer graphene. This feature is generally associated with multilayer graphene-like structures with weak interlayer coupling, consistent with a turbostratic stacking arrangement in which adjacent carbon layers are randomly oriented and lack long-range registry.^30^

When considered together with the XRD results, which exhibit a diffuse (002) reflection, the Raman spectrum is consistent with a predominantly disordered carbon framework composed of misaligned graphene-like layers. The coexistence of short-range ordering and structural disorder is a typical characteristic of activated carbon materials and is often associated with the development of surface heterogeneity.

#### 3.1.3. SEM-EDS

##### 3.1.3.1 SEM

**Fig. 5:**
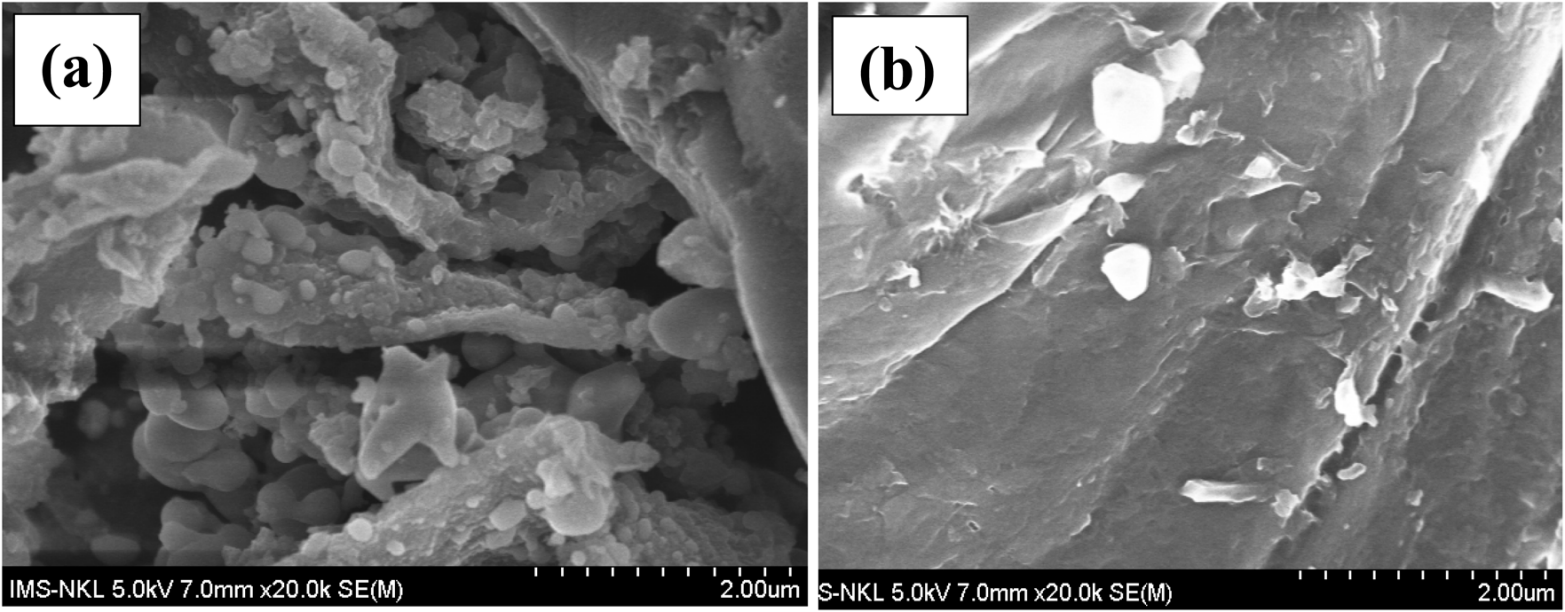
SEM images of pristine ACLDP (a) and ACLDP after MB adsorption (b).

The surface morphology of ACLDP before and after interaction with methylene blue (MB) was examined using scanning electron microscopy (SEM). Prior to adsorption (Fig. 5a), the material exhibits a heterogeneous texture composed of aggregated particles and irregular flake-like structures distributed without a defined orientation. The surface appears rough, with visible grooves and interparticle voids, reflecting a complex topography. Such features are commonly associated with porous carbon materials and may facilitate the accessibility of adsorbate molecules to the internal structure.

After exposure to MB (Fig. 5b), the surface morphology becomes comparatively smoother and more compact. Several grooves and rough features appear less distinct, suggesting partial coverage of the surface. This change is consistent with the presence of adsorbed species on the material surface and possible occupation of accessible pores. While SEM does not provide direct evidence of adsorption mechanisms, the observed morphological differences support the occurrence of interactions between MB and the ACLDP surface.

##### 3.1.3.2 EDS

**Fig. 6:**
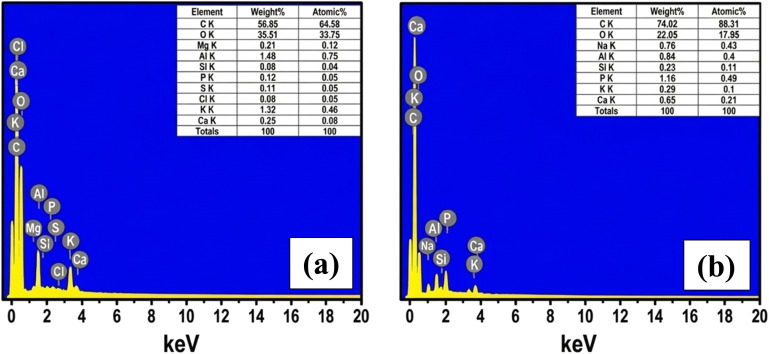
EDS spectra of LDP (a) and ACLDP (b).

Energy-dispersive X-ray spectroscopy (EDS) was employed to examine the elemental composition of the precursor (LDP) and the derived carbon material (ACLDP). For the raw LDP sample (Fig. 6a), the spectrum is dominated by carbon (56.85 wt%) and oxygen (39.51 wt%), reflecting the typical lignocellulosic nature of biomass, which is rich in oxygen-containing functional groups. Minor amounts of inorganic elements, including K, Ca, Mg, Al, Si, P, S, and Cl, are also detected, likely originating from naturally occurring mineral components in the biomass.

After hydrothermal treatment, chemical activation, and carbonization (Fig. 6b), the carbon content increases to 74.02 wt%, while the oxygen content decreases to 22.05 wt%. This compositional shift is consistent with the progressive removal of oxygen-containing groups and volatile components during thermal treatment, along with the development of a more carbon-rich framework. Trace elements such as Na, K, Ca, Al, Si, and P remain detectable, suggesting partial retention of inorganic species within the carbon matrix.

The observed changes in elemental composition are consistent with the transformation of biomass into a carbonaceous material with reduced oxygen content and increased aromatic character. Such evolution in composition is commonly discussed in relation to the formation of more stable carbon frameworks and is often associated with adsorption applications involving organic contaminants such as MB.

#### 3.1.4. Surface area and porosity (BET)

**Fig. 7:**
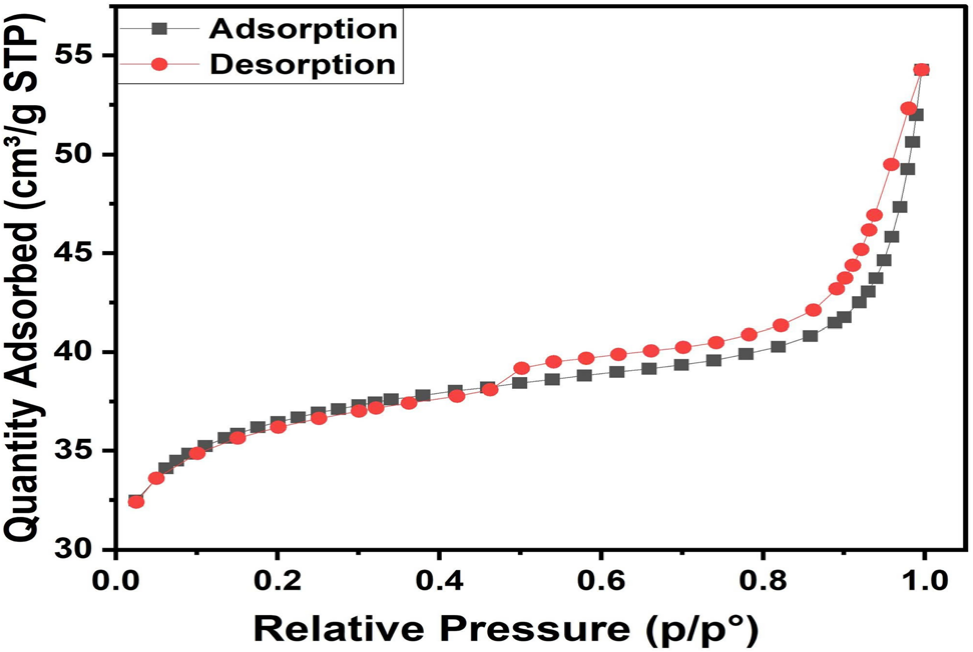
Nitrogen adsorption–desorption isotherm of ACLDP.

The textural properties of ACLDP were evaluated by nitrogen adsorption–desorption measurements at 77 K. The isotherm (Fig. 7) can be classified as type IV according to the IUPAC classification, with a noticeable hysteresis loop in the intermediate relative pressure range (*P*/*P*_0_ ≈ 0.4–0.9), which is typically associated with mesoporous structures.^32^

The specific surface area determined by the BET method is 115.12 m^2^ g^−1^. The total pore volume and average pore diameter, estimated using the BJH model, are 0.036 cm^3^ g^−1^ and 5.88 nm, respectively. The observed hysteresis loop may be related to H3/H4-type behavior, commonly reported for carbon materials with slit-like pores formed by the stacking or aggregation of carbon layers.

Such pore characteristics are commonly observed in biomass-derived carbons and are closely associated with adsorption behavior, as they can facilitate mass transport of adsorbate molecules within the pore network. Mesopores in this size range may provide accessible pathways for relatively large molecules, while the developed surface area offers sites for surface interactions.^18^

These structural features are therefore consistent with adsorption applications involving organic compounds such as dyes and antibiotics in aqueous systems, as reported in previous studies.^33^

These textural features are particularly important for interpreting adsorption behavior in heterogeneous porous carbons. The mesoporous network (∼5.88 nm) likely facilitates diffusion-assisted transport of MB molecules toward internal adsorption regions, while smaller pores contribute to adsorption-site availability. Consequently, adsorption in ACLDP cannot be adequately interpreted solely through surface-area metrics, because pore accessibility and transport behavior jointly influence adsorption performance.

The coexistence of transport-accessible mesopores and structurally heterogeneous adsorption domains may also explain why multiple adsorption models later exhibit statistically comparable fitting performance before AIC discrimination is applied.

#### 3.1.5. Thermal behavior and stability (TGA)

**Fig. 8:**
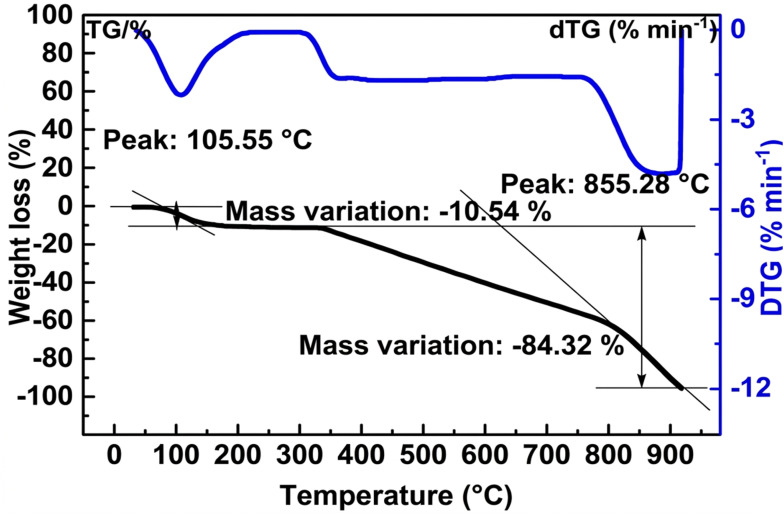
Thermogravimetric analysis of ACLDP.

The thermogravimetric (TG/DTG) profile of ACLDP (Fig. 8) reflects the thermal decomposition behavior commonly observed in carbon materials derived from lignocellulosic biomass. An initial mass loss occurs below approximately 150 °C, with a DTG peak near 105 °C and a weight reduction of about 10.54%. This stage is generally associated with the removal of physically adsorbed water and residual volatile species retained within the pore structure.

In the temperature range of 200–600 °C, a gradual mass loss is observed, which can be attributed to the thermal decomposition of lignocellulosic components such as hemicellulose, cellulose, and lignin. These constituents decompose over partially overlapping temperature intervals, leading to a continuous decrease in mass and reflecting the progressive transformation of the precursor into a carbonaceous framework.

At higher temperatures (≈800–900 °C), a more pronounced mass loss is detected, with a DTG peak around 855 °C (total mass loss reaching approximately 84.32%). This stage may be related to the further decomposition or rearrangement of the remaining carbon structure, including the breakdown of more thermally stable domains.

The overall thermal profile suggests that ACLDP undergoes staged decomposition typical of biomass-derived carbons and retains a fraction of thermally stable carbon at elevated temperatures. Such behavior is commonly reported for porous carbon materials produced from agricultural residues through carbonization and activation processes.^34,35^

#### 3.1.6. Point of zero charge (pH_pzc_)

**Fig. 9:**
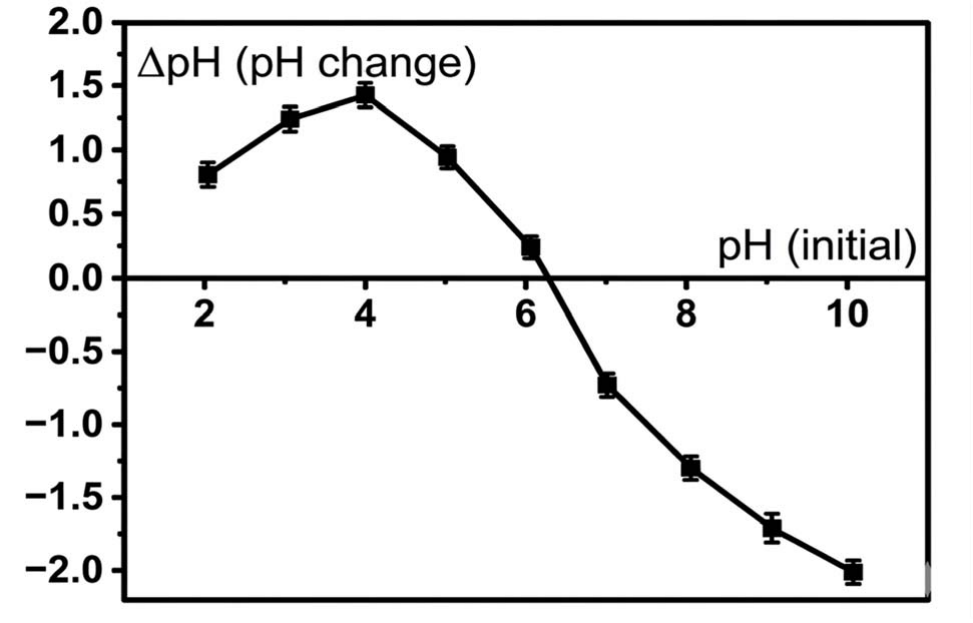
Determination of the point of zero charge (pH_pzc_) of ACLDP.

The point of zero charge (pH_pzc_) of ACLDP was evaluated using the pH drift method, and the corresponding relationship between ΔpH and initial pH is presented in Fig. 9. The ΔpH curve intersected the zero line at approximately pH 6.3, indicating the pH region where proton-consuming and proton-releasing surface reactions became nearly balanced.

At pH values lower than 6.3, positive ΔpH values were observed, reflecting progressive protonation of surface functional groups under acidic conditions. In contrast, negative ΔpH values obtained above pH 6.3 were associated with increasing surface deprotonation and the gradual formation of negatively charged surface sites. Such pH-responsive behavior is characteristic of activated carbons enriched with oxygen-containing surface functionalities.

#### 3.1.7. Zeta potential

**Fig. 10:**
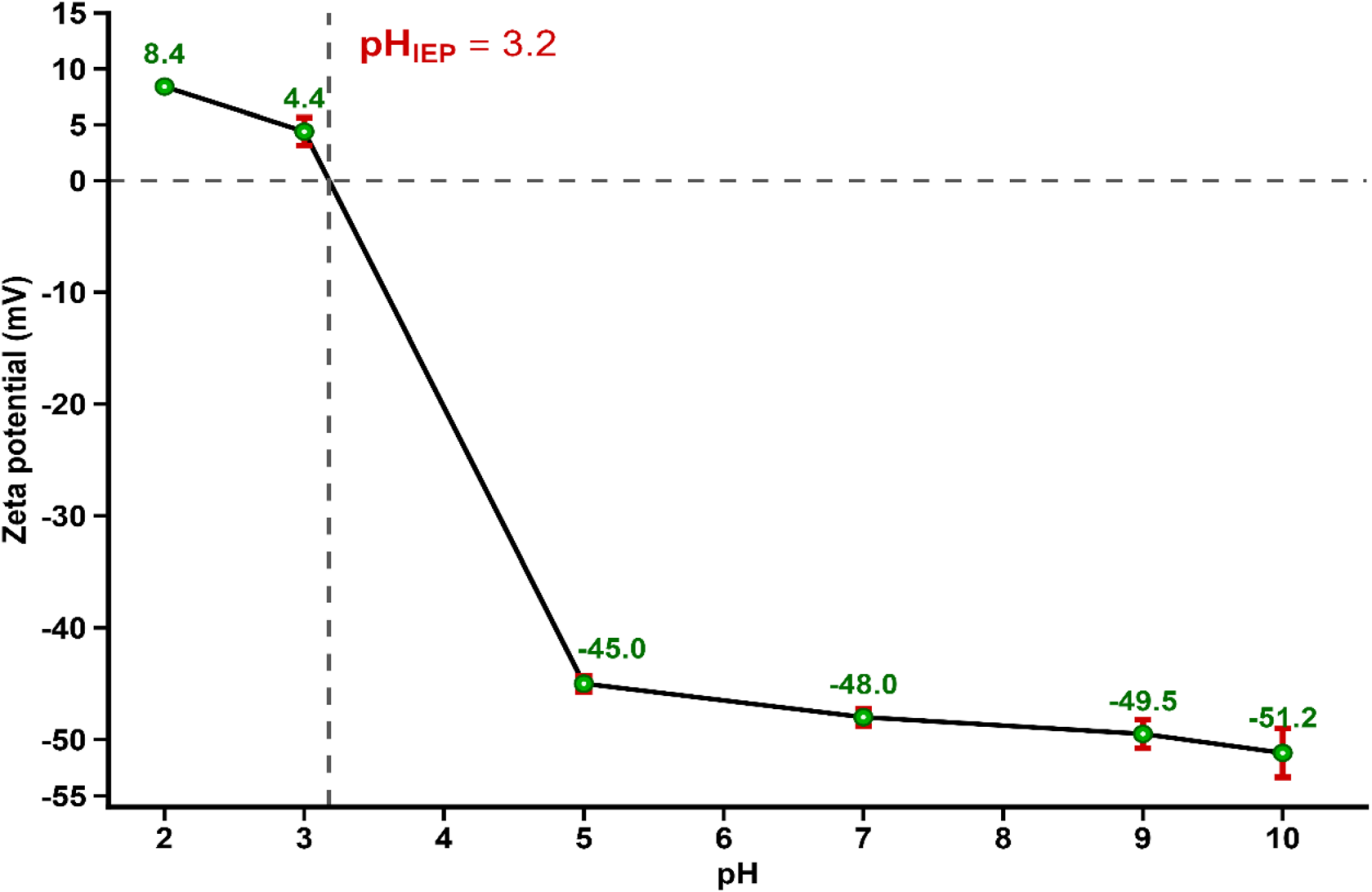
Zeta potential of ACLDP as a function of solution pH, showing the isoelectric point (pH_IEP_) at approximately 3.2. Error bars represent standard deviation from triplicate measurements.

Additional insight into the electrokinetic behavior of ACLDP was obtained from zeta potential measurements performed across a broad pH range (Fig. 10). The zeta potential shifted progressively from +8.4 ± 0.03 mV at pH 2 to −51.2 ± 2.19 mV at pH 10. The zeta potential crossover occurred near pH 3.2, corresponding to the isoelectric point (pH_IEP_).

Above the pH_IEP_, ACLDP exhibited increasingly negative electrokinetic potential values, consistent with progressive deprotonation of oxygen-containing surface functional groups. This trend reflects the progressive development of negatively charged surface regions together with increasing electrostatic attraction toward cationic methylene blue (MB^+^) species. The pronounced decrease in zeta potential under neutral and alkaline conditions further supports the formation of negatively charged adsorption regions on the carbon surface.

The pH_pzc_ value derived from the pH drift method differed from the isoelectric point obtained from zeta potential analysis, a phenomenon frequently reported for heterogeneous porous carbons. The pH drift method reflects the overall acid–base behavior of the bulk adsorbent surface, whereas zeta potential measurements characterize the electrokinetic potential at the slipping plane of dispersed particles. Therefore, the separation between pH_pzc_ (∼6.3) and pH_IEP_ (∼3.2) is consistent with the structurally heterogeneous nature of activated carbon materials containing diverse oxygenated functional groups and hierarchical pore networks. Similar observations have been reported for other porous carbonaceous adsorbents, where differences between pH_pzc_ and pH_IEP_ were attributed to heterogeneous surface charge distribution and non-uniform electrokinetic behavior within porous structures.^36,37^

Taken together, the pH_pzc_ and zeta potential analyses support an important contribution of electrostatic interactions during MB adsorption, particularly under neutral and alkaline conditions where ACLDP exhibits strongly negative surface potential values favorable for interaction with cationic MB species. Nevertheless, electrostatic attraction alone is unlikely to fully govern the adsorption process, and additional interactions including π–π stacking and hydrogen bonding are also likely to contribute cooperatively to MB uptake. The zeta potential results are also consistent with the observed enhancement of MB adsorption under neutral and alkaline conditions.

#### 3.1.8. Physicochemical properties and iodine number of ACLDP

The physicochemical properties of ACLDP are summarized in Table 2.

**Table 2:** Physicochemical properties of ACLDP

Parameter	ACLDP
Ash content (%)	1.46 ± 0.05
Moisture content (%)	2.68 ± 0.05
Bulk density (g cm^−3^)	0.68 ± 0.02

The measured values fall within the range typically observed for biomass-derived activated carbons.^38^ These parameters are frequently considered when assessing the handling characteristics and practical applicability of carbon materials in adsorption-related systems.

In addition, the iodine number of ACLDP, determined to be 812 mg g^−1^, provides further insight into its textural properties. The iodine number is widely used as an indicator related to the micropore content of activated carbons. The obtained value lies within the range typically reported for materials used in practical applications (approximately 500–1200 mg g^−1^), suggesting a hierarchical micro–mesoporous structure.

Such a value is commonly associated with the presence of a developed microporous structure and is often discussed in relation to adsorption processes involving relatively small molecules.

### 3.2. Effect of operating parameters on MB adsorption by ACLDP

To ensure reproducibility, each adsorption experiment was conducted in triplicate under identical conditions, and the reported values correspond to the average of three measurements.

#### 3.2.1. Effect of pH

**Fig. 11:**
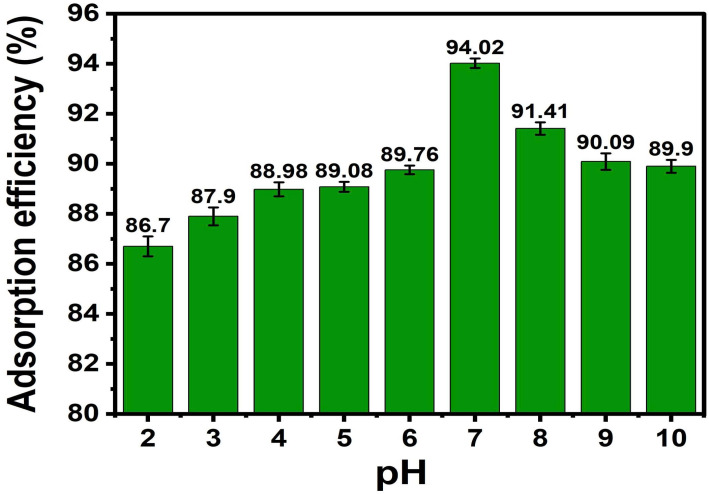
Effect of pH on MB adsorption efficiency.

A mass of 0.03 g of ACLDP was added to a 100 mL conical flask containing 25 mL of MB solution with an initial concentration of 50.00 mg L^−1^. The solution pH was adjusted in the range of 3–10 using 0.1 M HCl or 0.1 M NaOH. The suspensions were agitated at 300 rpm for 90 min at room temperature (25 ± 1 °C). After equilibration, the mixtures were centrifuged at 4000 rpm for 15 min to separate the solid phase, and the residual MB concentration in the supernatant was determined. The corresponding results are presented in Fig. 11 and S2 (SI).

The adsorption behavior varies with solution pH. The highest removal efficiency, 94.02%, was obtained at pH 7, while the point of zero charge (pH_pzc_) of ACLDP is 6.3. At pH values below pH_pzc_, the surface tends to be positively charged due to protonation of surface functional groups, which may reduce electrostatic attraction with cationic MB species (MB^+^). As the pH increases above pH_pzc_, deprotonation of oxygen-containing groups such as –COOH and –OH can generate negatively charged sites (–COO^−^, –O^−^), which are commonly associated with stronger electrostatic interactions with MB^+^.

At higher pH values, the presence of excess OH^−^ ions in solution may influence the interaction between MB and the adsorbent surface. Under near-neutral conditions (pH ≈ 7), electrostatic interactions are likely favorable. In addition, π–π interactions between the aromatic structure of MB and graphitic domains in ACLDP may also contribute to adsorption.^39^

This behavior is consistent with previous studies on MB adsorption using activated carbon and biomass-derived carbon materials, where near-neutral pH conditions are often reported as suitable for adsorption processes.^40–42^ Based on these observations, pH 7 was selected for subsequent adsorption experiments.

#### 3.2.2. Contact time and adsorption equilibrium

**Fig. 12:**
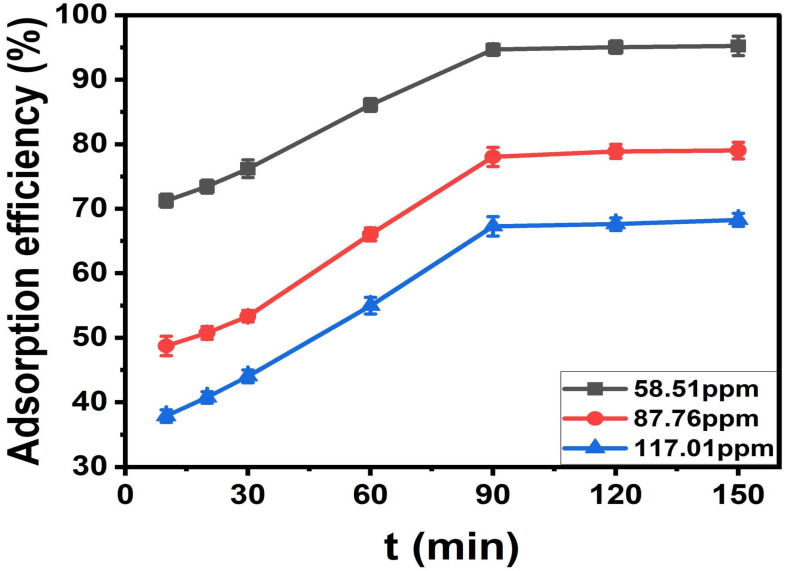
Effect of contact time on MB adsorption efficiency.

A mass of 0.03 g of ACLDP was added to a 100 mL conical flask containing 25 mL of MB solutions with initial concentrations of 58.00, 87.00, and 117.00 mg L^−1^. The pH of all solutions was adjusted and maintained at 7. The adsorption process was carried out under agitation at 300 rpm for different contact times (10, 20, 30, 60, 90, 120, and 150 min) at room temperature (25 ± 1 °C). At predetermined intervals, samples were withdrawn and centrifuged at 4000 rpm to separate the solid phase. The supernatant was then collected using a micropipette for determination of the residual MB concentration. The corresponding results are presented in Fig. 12 and S3 (SI). The adsorption efficiency increases with contact time for all investigated initial concentrations (58.51, 87.76, and 117.01 mg L^−1^). A rapid increase is observed during the initial stage (10–30 min), particularly at the lower concentration. This behavior may be associated with the availability of accessible adsorption sites on the ACLDP surface, allowing interactions between MB molecules and the adsorbent to occur more readily. As contact time progresses, the rate of adsorption gradually decreases, and the system approaches equilibrium within approximately 90–120 min. This trend is commonly attributed to the progressive occupation of available surface sites, along with the increasing contribution of diffusion-related processes. Similar time-dependent adsorption behavior has been reported for porous carbon materials in aqueous systems.^43^ Based on these observations, a contact time of 90 min was selected for subsequent adsorption experiments.

#### 3.2.3. Effect of adsorbent dosage

**Fig. 13:**
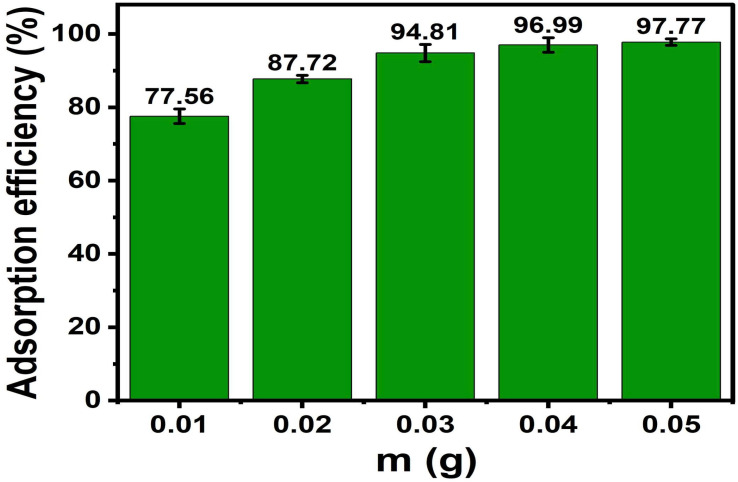
Effect of adsorbent dosage on MB adsorption efficiency.

ACLDP with varying dosages (0.01–0.05 g) was added to 100 mL conical flasks. Each flask contained 25 mL of MB solution with an initial concentration of 50.00 mg L^−1^, and the solution pH was adjusted and maintained at 7. The adsorption experiments were conducted under agitation at 300 rpm for 90 min at room temperature (25 ± 1 °C). After completion, the suspensions were centrifuged at 4000 rpm for 15 min to separate the solid phase, and the residual MB concentration in the supernatant was determined. The results are presented in Fig. 13 and S4 (in SI).

The removal efficiency (*H*%) increases as the ACLDP dosage rises from 0.01 to 0.05 g, with values ranging from 77.56% to 97.77%. This trend may be associated with the increase in available surface area and the number of accessible adsorption sites as the amount of adsorbent increases, allowing more MB molecules to interact with the material surface.^44^ At lower dosages, the number of available sites is comparatively limited, which corresponds to lower removal efficiency.^45^

When the dosage increases from 0.03 to 0.05 g, the change in removal efficiency becomes less pronounced, suggesting that the system approaches a saturation condition under the given experimental setup. At higher dosages, particle aggregation or partial overlap of adsorption sites may occur, which can reduce the effective surface area and limit access of MB molecules to available sites.

Based on these observations, a dosage of 0.03 g per 25 mL (equivalent to 1.2 g L^−1^) was selected for subsequent adsorption experiments.

#### 3.2.4. Effect of temperature

**Fig. 14:**
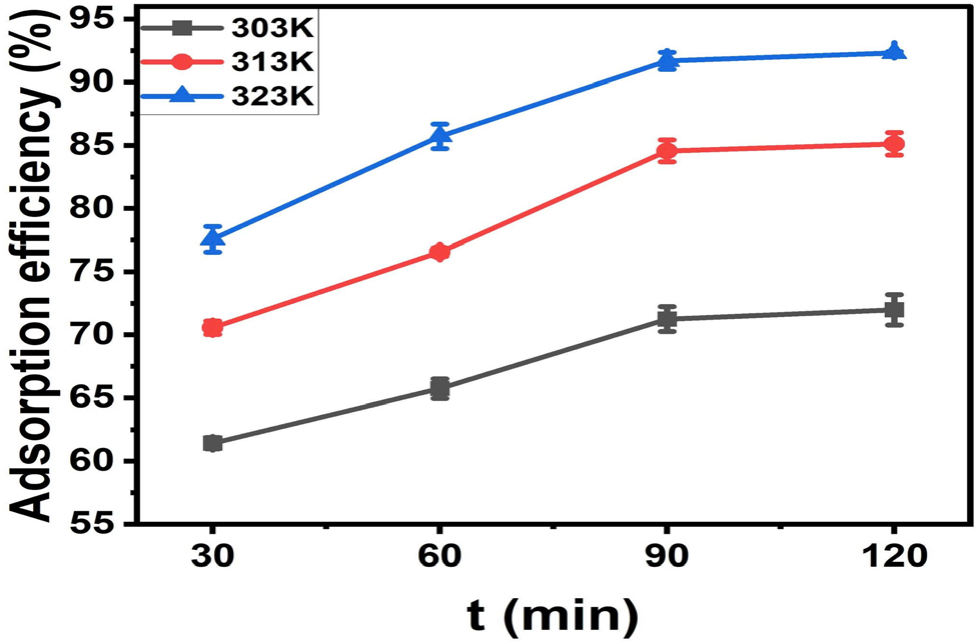
Effect of temperature on MB adsorption efficiency.

A series of 100 mL conical flasks were prepared, each containing 0.03 g of ACLDP and 25 mL of MB solution with an initial concentration of 100.00 mg L^−1^. The solution pH was adjusted to 7 using 0.1 M NaOH and 0.1 M HNO_3_. Adsorption experiments were conducted under stirring at 300 rpm using a thermostatic magnetic stirrer at temperatures of 303, 313, and 323 K for contact times ranging from 30 to 120 min. After adsorption, the suspensions were centrifuged to separate the solid phase, and the residual MB concentration in the supernatant was determined. The results are presented in Fig. 14 and S5 (SI).

An increase in temperature is accompanied by an increase in MB removal efficiency under the studied conditions. This trend is commonly attributed to enhanced mass transfer at elevated temperatures. Higher temperature can increase molecular mobility and reduce solution viscosity, which may facilitate the transport of MB molecules from the bulk solution to the adsorbent surface.

In addition, temperature may influence diffusion-related processes within the pore structure, potentially improving access of MB molecules to internal adsorption sites. Similar effects have been observed for porous carbon materials in aqueous adsorption systems.^46^

Previous studies have also examined the thermodynamic parameters of MB adsorption on carbon-based materials, where positive enthalpy changes (Δ*H*° > 0) have been reported in some cases, suggesting endothermic behavior.^47^ However, confirmation of thermodynamic nature requires dedicated analysis based on equilibrium data. The observed temperature dependence in the present study is therefore discussed in terms of transport and accessibility effects under the experimental conditions.

#### 3.2.5. Effect of initial MB concentration

**Fig. 15:**
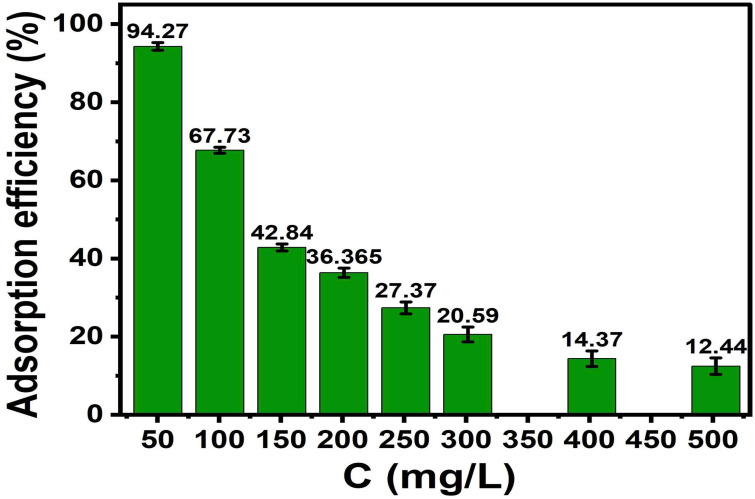
Effect of initial concentration on MB adsorption efficiency.

A series of 100 mL conical flasks were prepared, each containing 0.03 g of ACLDP and 25 mL of MB solution with initial concentrations ranging from 50 to 500 mg L^−1^. The solution pH was adjusted and maintained at 7. Adsorption experiments were conducted under agitation at 300 rpm for 90 min at room temperature (25 ± 1 °C). After completion, the suspensions were centrifuged at 4000 rpm for 15 min. The supernatant was collected using a micropipette, and the residual MB concentration was determined. The corresponding results are presented in Fig. 15 and S6 (in SI).

The removal efficiency varies with the initial MB concentration. At lower concentrations, the number of MB molecules in solution is relatively small compared to the available adsorption sites, and higher removal efficiency is observed. As the initial concentration increases from 50 to 500 mg L^−1^, the number of MB molecules increases while the number of available adsorption sites remains limited, which is associated with a decrease in removal efficiency (from 94.27% to 12.44%).

At the same time, increasing the initial concentration leads to a higher concentration gradient between the bulk solution and the adsorbent surface, which may facilitate mass transfer and is often discussed in relation to increased adsorption capacity. Similar trends have been reported for dye adsorption on carbon-based materials.^47^

### 3.3. Adsorption isotherm modeling and statistical evaluation

Adsorption isotherm models provide useful insight into surface characteristics and interactions between the adsorbate and the adsorbent. Based on experimental data obtained from the effect of initial MB concentration on removal efficiency and adsorption capacity, several isotherm models were applied to describe the adsorption process.

Classical models, including Langmuir, Freundlich, and Temkin, were considered alongside extended models such as Dubinin–Radushkevich, Sips, Toth, Elovich, and Halsey. All models were fitted in their nonlinear forms to the experimental data.

The use of nonlinear fitting helps avoid potential distortions associated with linearization and allows the parameters to be estimated in a manner more consistent with the original model formulations.^48^

**Fig. 16:**
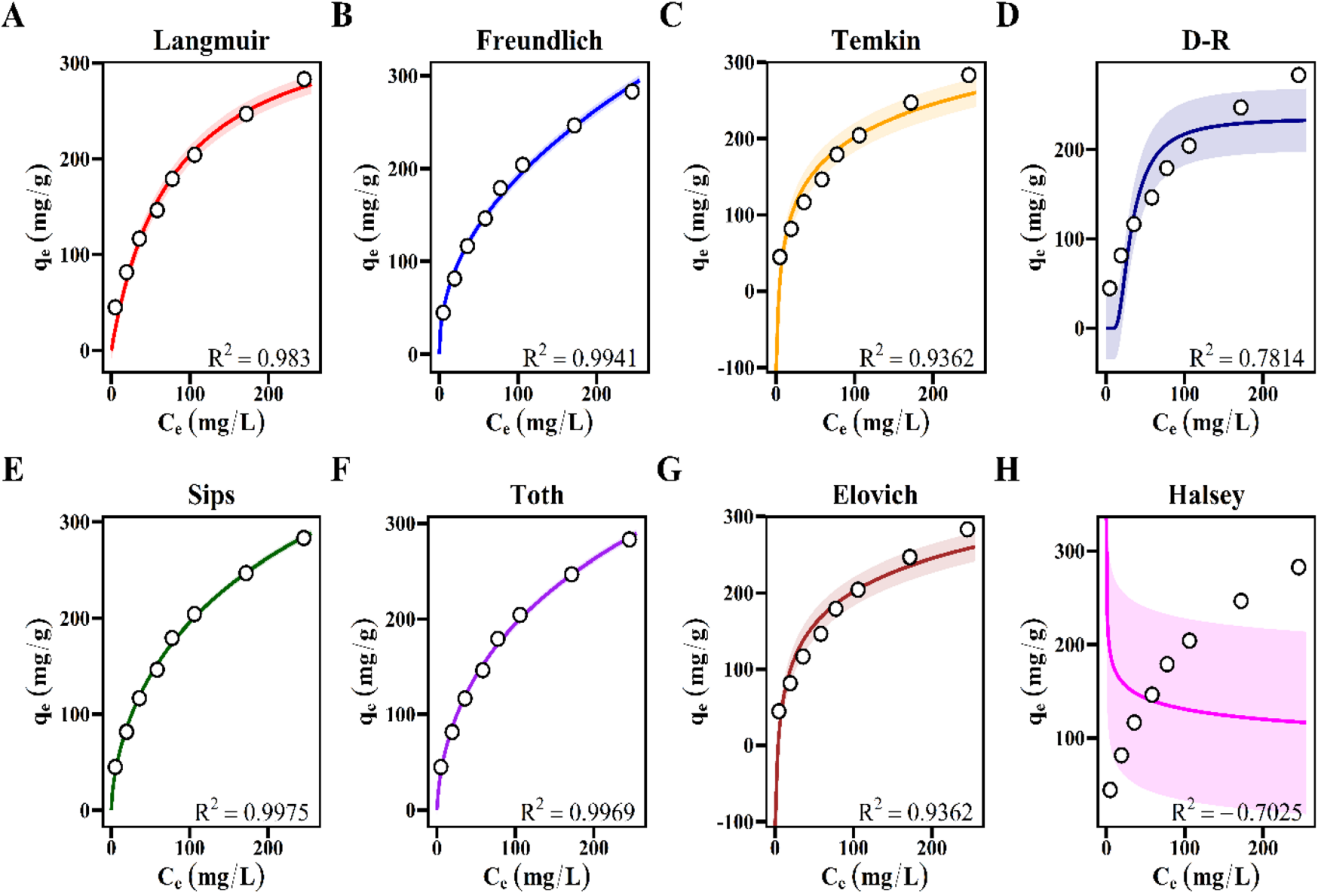
Nonlinear isotherm plots of *q*_e_*versus C*_e_ for MB adsorption on ACLDP fitted with the (A) Langmuir, (B) Freundlich, (C) Temkin, (D) Dubinin–Radushkevich (D–R), (E) Sips, (F) Toth, (G) Elovich, and (H) Halsey isotherm models.

The relationship between equilibrium adsorption capacity (*q*_e_) and equilibrium concentration (*C*_e_) was analyzed using nonlinear isotherm models. These include Langmuir, Freundlich, Temkin, Dubinin–Radushkevich, Sips, Toth, Elovich, and Halsey. The fitted curves are presented in Fig. 16, and the corresponding parameters are listed in Table 3.

**Table 3:** Nonlinear isotherm parameters for MB adsorption on ACLDP

Model	Parameters	Values
Langmuir	*q*_max_ (mg g^−1^)	≈345.8
	*K*_L_ (L mg^−1^)	≈0.0063
Freundlich	*K*_f_ ((mg g^−1^) (L mg^−1^)^1/*n*^)	≈28.7
	*n*	≈2.21
Temkin	*A*_T_ (L g^−1^)	≈0.92
	*B*_T_ (J mol^−1^)	≈58.4
Sips	*q*_max_ (mg g^−1^)	≈351.2
	*K*_s_ (L mg^−1^)	≈0.0058
Toth	*q*_max_ (mg g^−1^)	≈348.5
	*K*_*t*_ (L mg^−1^)	≈0.0061
Halsey	*K*_H_	≈0.018
	*n*	≈0.62
Elovich	*Q*_E_ (mg g^−1^)	≈310.4
	*K*_E_ (g mg^−1^)	≈0.021
Dubinin–Radushkevich	*q*_max_ (mg g^−1^)	≈290.7
	*B* (mol^2^ kJ^−2^)	≈1.45 × 10^−6^

Model parameters include *q*_max_ and affinity constants for each model. For example, Langmuir gives *q*_max_ ≈ 345.8 mg g^−1^ and *K*_L_ ≈ 0.0063 L mg^−1^. The Sips and Toth models provide comparable *q*_max_ values, near 350 mg g^−1^. Other models yield parameters consistent with heterogeneous adsorption systems.

Model performance was evaluated using RMSE, AIC, ΔAIC, and the coefficient of determination (*R*^2^). The best-performing model corresponds to the lowest RMSE and AIC values, along with the highest *R*^2^. The statistical results are summarized in Table 4.

**Table 4:** Statistical metrics and ranking of isotherm models

Model	AIC	*R* ^2^	RMSE (mg g^−1^)	ΔAIC	Ranking
**Sips**	**52.17**	**0.9975**	**3.83**	**0.00**	**1**
Toth	53.86	0.9969	4.25	1.69	2
Freundlich	57.04	0.9941	5.88	4.87	3
Langmuir	65.45	0.9830	9.94	13.28	4
Temkin	76.03	0.9362	19.26	23.86	5
Elovich	76.03	0.9362	19.26	23.86	5
D–R	85.88	0.7814	35.64	33.71	6
Halsey	102.30	−0.7025	99.46	50.13	7

The Sips model exhibited the best overall statistical performance, with the highest coefficient of determination (*R*^2^ = 0.9975), the lowest RMSE value (3.83 mg g^−1^), and the lowest AIC value (52.17), resulting in a ΔAIC value of 0. The Toth model also showed strong agreement with the experimental data (*R*^2^ = 0.9969) and remained statistically competitive with the Sips model (ΔAIC = 1.69). According to Akaike information-theoretic interpretation, models with ΔAIC < 2 may be considered statistically comparable. Under this condition, both Sips and Toth models provide similarly plausible descriptions of the adsorption system.

Importantly, the present results demonstrate that reliance solely on correlation coefficients may lead to ambiguous mechanistic interpretation in heterogeneous porous carbon systems. Several models, including Sips, Toth, and Freundlich, produced similarly high *R*^2^ values despite representing different adsorption assumptions and surface-energy distributions. The incorporation of AIC therefore provides additional discriminatory power by simultaneously considering fitting accuracy and model complexity, thereby reducing the risk of mechanistic overinterpretation associated with overparameterized models.

The statistically preferred performance of the Sips model suggests that MB adsorption onto ACLDP occurs within a structurally heterogeneous adsorption environment while progressively approaching saturation behavior at elevated concentrations. This interpretation is physically consistent with the hierarchical porous structure and heterogeneous surface chemistry identified from BET, FTIR, Raman, and electrokinetic analyses. At lower equilibrium concentrations, adsorption behavior follows a Freundlich-like tendency associated with energetically heterogeneous adsorption sites. As concentration increases, the adsorption process gradually approaches Langmuir-type saturation behavior due to progressive occupation of accessible adsorption regions within the hierarchical pore network.^49^

The comparatively weaker performance of the Langmuir model (*R*^2^ = 0.9830; RMSE = 9.94 mg g^−1^; ΔAIC = 13.28) further suggests that the assumption of energetically uniform monolayer adsorption is insufficient to fully represent the ACLDP system. This limitation is likely associated with the structurally heterogeneous nature of biomass-derived porous carbons, where defect-rich graphitic domains, oxygen-containing functional groups, and irregular pore structures collectively generate a broad distribution of adsorption energies.^49,50^

The Freundlich model also supports this interpretation because it is commonly associated with adsorption systems exhibiting non-uniform adsorption energies distributed across heterogeneous surfaces. Meanwhile, the moderate performance of Temkin, Elovich, and Dubinin–Radushkevich models suggests that no single simplified adsorption-energy assumption can completely capture the complexity of the present adsorption system. In contrast, the Halsey model exhibited poor statistical performance, including a negative *R*^2^ value and substantially higher RMSE and AIC values, indicating limited applicability under the investigated conditions.

Taken together, these observations indicate that adsorption behavior in ACLDP cannot be adequately interpreted using a purely homogeneous adsorption mechanism or through surface-area considerations alone. Instead, the adsorption process likely involves the coupled contribution of heterogeneous surface interactions, hierarchical pore accessibility, diffusion-assisted transport, and progressive occupation of adsorption sites with different energy distributions. From Table 3, the maximum adsorption capacity (*q*_max_) of ACLDP is estimated to be approximately 345.8 mg g^−1^.

The present statistical analysis further highlights the importance of combining information-theoretic criteria with conventional error-function analysis when interpreting adsorption behavior in heterogeneous biomass-derived carbons. Under complex adsorption conditions involving overlapping transport and surface-interaction pathways, multiple models may provide statistically acceptable fitting performance despite representing fundamentally different physical mechanisms. The integration of AIC therefore improves mechanistic discrimination by identifying models that achieve a more physically meaningful balance between fitting accuracy and parameter complexity.

#### 3.3.1. Comparison *q*_max_ with other biomass-derived carbon materials

A comparison of the maximum adsorption capacity of ACLDP with other biomass-derived carbon materials is presented in Table 5. The *q*_max_ value of ACLDP is higher than that reported for many biochars, conventional activated carbons, and modified carbon materials.

**Table 5:** Comparison of maximum adsorption capacity (*q*_max_) for MB on biomass-derived carbon materials^a^

Adsorbent material	Precursor	*q*_max_ (mg g^−1^)	Raw material cost	Synthesis complexity & preparation time	Kinetic equilibrium time (*t*_eq_)	Cost-effectiveness evaluation	Ref.
Biochar	Jackfruit peel	39.87	Low-cost	Simple (pyrolysis, few hours)	∼60–120 min	Low (low *q* but inexpensive)	55
Activated carbon	Commercial granular activated carbon	240	High-cost	Not self-synthesized	∼30–60 min	Moderate (high performance but expensive)	56
Ball-milled biochar	Rice straw	50.27	Low-cost	Moderate (mechanical milling required)	∼60–120 min	Moderate	57
Activated carbon	Moringa leaves	136.99	Relatively low-cost	Moderate (activation required)	∼60–120 min	Good	40
Activated carbon	Durian peel	57.47	Low-cost	Moderate	∼60–120 min	Moderate	42
Activated carbon	Durian peel and seeds	137	Low-cost	Moderate	∼60–120 min	Good	41
Activated carbon	Lignocellulosic waste	148.8	Relatively low-cost	Moderate	∼30–90 min	Good	58
Activated carbon	Non-wood agricultural residues	85.2	Low-cost	Moderate	∼60–120 min	Fair	59
Biochar	Mushroom cultivation waste	∼322	Low-cost	Simple	∼60–120 min	Excellent	60
Biochar	Oil palm bunch residues	∼218	Low-cost	Simple	∼60–120 min	Good	61
Fe_3_O_4_-N doped biochar	Banana peel	312.5	Moderate-cost	Complex (modification + nanomaterial incorporation)	∼30–60 min	Good but costly	11
Activated carbon (ACLDP)	*Lansium domesticum* peel	345.8	Low-cost	Moderate (activation required)	∼60–120 min	Good	This study

a The cost evaluation presented in this table is qualitative and based on precursor availability, synthesis complexity, chemical consumption, and energy requirements reported in the literature rather than detailed economic analysis.

This observation may be associated with the development of pore structure and the presence of surface functional groups formed during carbonization, hydrothermal treatment, and H_3_PO_4_ activation. ACLDP achieved a methylene blue adsorption capacity of approximately 345.8 mg g^−1^ despite exhibiting a moderate BET surface area of 115.12 m^2^ g^−1^. Comparable behavior has been reported for several biomass-derived carbon adsorbents, indicating that adsorption capacity does not scale directly with BET surface area alone. Magnolia leaf biochar with a BET surface area of only 41.8 m^2^ g^−1^ adsorbed approximately 114 mg g^−1^ of MB,^51^ whereas Mn-modified lignin biochar with a surface area near 96 m^2^ g^−1^ achieved adsorption capacities around 249 mg g^−1^.^52^ Elephant dung biochar also exhibited MB uptake close to 150 mg g^−1^ despite possessing a relatively low surface area of ∼32 m^2^ g^−1^.^53^ In another report, a fly ash/biochar composite with a BET surface area near 73 m^2^ g^−1^ adsorbed more than 800 mg g^−1^ of MB.^54^ These comparisons suggest that BET surface area alone cannot adequately account for MB adsorption behavior in heterogeneous porous carbon systems.

The relatively high adsorption performance of ACLDP is more likely associated with the combined influence of pore accessibility, mesoporous diffusion pathways, and surface chemistry. Methylene blue molecules are relatively large (∼1.43 nm), and access to ultramicropores may become restricted in carbons dominated by narrow pore channels.^49^ Under such conditions, a high BET surface area does not necessarily translate into proportionally higher adsorption capacity because part of the measured surface may remain inaccessible to MB molecules. ACLDP exhibited an average pore diameter of approximately 5.88 nm, which falls within the mesoporous range and is favorable for diffusion-assisted transport toward internal adsorption regions within the hierarchical carbon framework. Surface functionality also appears to contribute substantially to adsorption performance. Oxygen-containing groups such as –OH and –COOH can provide adsorption sites capable of electrostatic interactions and hydrogen bonding with cationic dye molecules.^62^ In addition, conjugated aromatic carbon domains may strengthen adsorption through π–π interactions between the graphitic surface and the aromatic structure of methylene blue.^20^ Similar cooperative effects involving pore accessibility, mesoporous diffusion, surface functionality, and π–π interactions have also been reported for other biomass-derived carbon adsorbents used in MB removal.^20,49,50^ Accordingly, the adsorption performance of ACLDP likely results from the interplay between accessible hierarchical pore structure and chemically active surface domains rather than from surface area alone.

### 3.4. Kinetic adsorption

The adsorption kinetics of MB onto ACLDP were evaluated using five commonly applied kinetic models, including pseudo-first-order (PFO), pseudo-second-order (PSO), Elovich, Avrami, and Weber–Morris models. All models were fitted using their nonlinear forms to avoid distortions in error structure caused by linearization, thereby providing more reliable parameter estimation and mechanistic interpretation.

**Fig. 17:**
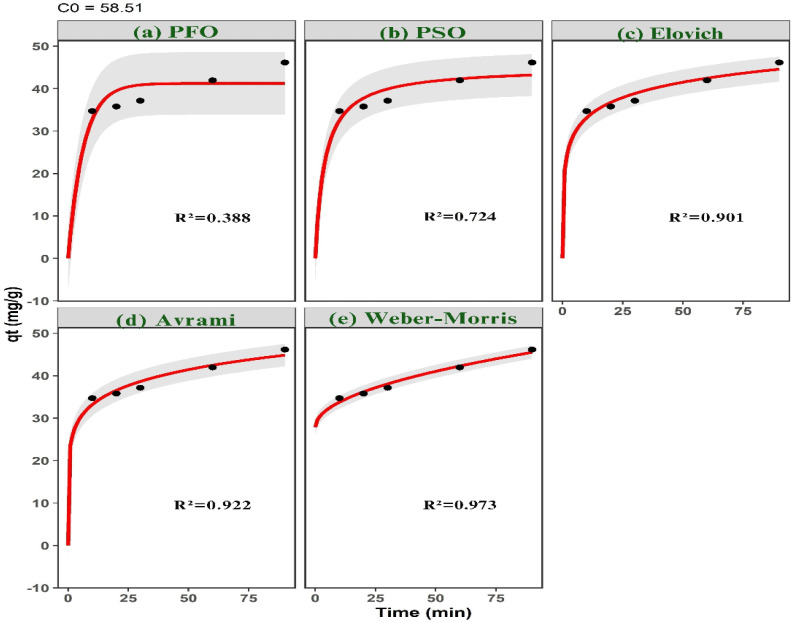
*q*_*t*_ *versus t* plots based on kinetic models at *C*_0_ = 58.51 mg L^−1^ fitted with the (a) pseudo-first-order (PFO), (b) pseudo-second-order (PSO), (c) Elovich, (d) Avrami, and (e) Weber–Morris kinetic models.

**Fig. 18:**
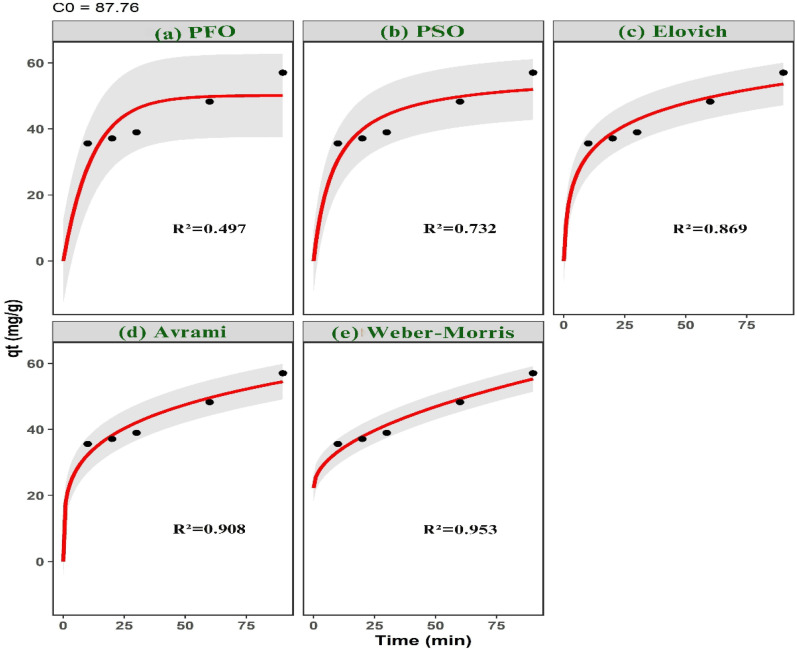
*q_t_ versus t* plots based on kinetic models at *C*_0_ = 87.76 mg L^−1^ fitted with the (a) pseudo-first-order (PFO), (b) pseudo-second-order (PSO), (c) Elovich, (d) Avrami, and (e) Weber–Morris kinetic models.

**Fig. 19:**
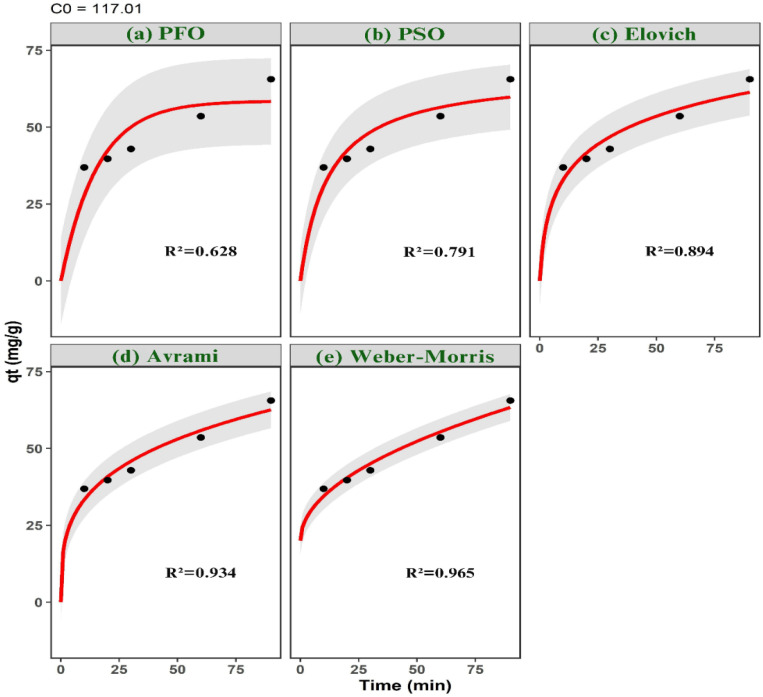
*q_t_ versus t* plots based on kinetic models at *C*_0_ = 117.01 mg L^−1^ fitted with the (a) pseudo-first-order (PFO), (b) pseudo-second-order (PSO), (c) Elovich, (d) Avrami, and (e) Weber–Morris kinetic models.

The variation of adsorption capacity (*q*_*t*_) with contact time (*t*) at different initial concentrations is illustrated in Fig. 17–19.

The statistical comparison of the kinetic models based on *R*^2^, RMSE, *χ*^2^, and AIC values (Table 6) demonstrates that adsorption kinetics in the ACLDP system cannot be adequately represented by a single homogeneous adsorption pathway. Although several kinetic models exhibited moderate-to-good fitting performance, substantial differences emerged after combined evaluation using error-function analysis and information-theoretic discrimination.

**Table 6:** Statistical comparison of kinetic models using *R*^2^, RMSE, *χ*^2^, AIC, and ΔAIC at different initial concentrations (*C*_0_)

*C*_0_ (mg L^−1^)	Kinetic model	*k*	*R* ^2^	RMSE (mg g^−1^)	*χ* ^2^	AIC
**58.51**	PFO	0.0960	0.388	4.0504	3.0791	23.5835
	PSO	0.0047	0.724	2.1303	0.8199	14.5882
	**Elovich**	344.8894	0.901	1.1767	0.2483	6.27897
	Avrami	0.1615	0.922	1.7064	0.5177	13.4819
	**Weber–Morris**	1.4942	0.973	1.1417	0.2074	5.8558
**87.76**	PFO	0.0538	0.497	5.8618	7.6904	28.7584
	PSO	0.0019	0.732	4.0435	2.6316	23.5595
	Elovich	24.1956	0.869	2.5485	1.0887	17.0975
	Avrami	0.063205	0.908	3.2907	1.7877	22.6756
	**Weber–Morris**	2.8885	0.953	2.2475	0.6961	15.3379
**117.01**	PFO	0.0449	0.628	6.2902	8.7968	29.7460
	PSO	0.0014	0.791	4.8111	3.2452	25.9929
	Elovich	14.75	0.894	2.9694	1.3273	19.2371
	Avrami	0.0492	0.934	3.7916	2.1713	24.6593
	**Weber–Morris**	3.7577	0.965	2.7399	0.8835	18.1110

Among the investigated models, the Weber–Morris model consistently produced the highest *R*^2^ values (0.953–0.973) together with the lowest RMSE, *χ*^2^, and AIC values across all investigated concentrations. The statistically preferred performance of the Weber–Morris model indicates that diffusion-assisted transport plays a dominant role in governing adsorption kinetics under the investigated conditions. Importantly, this observation should not be interpreted as evidence that intraparticle diffusion acts as the sole rate-limiting mechanism. Instead, the results suggest that adsorption proceeds through coupled transport pathways involving external mass transfer, pore diffusion, and surface interactions operating simultaneously within the heterogeneous porous structure.^63^

The Avrami model also exhibited strong agreement with the experimental data (*R*^2^ = 0.908–0.934), further supporting the interpretation that adsorption occurs through complex and multi-step kinetic pathways rather than through a single elementary adsorption mechanism. Such behavior is physically consistent with structurally heterogeneous porous carbons containing adsorption regions with different transport accessibility and surface-energy distributions.

The Elovich model also provides moderate-to-good fitting performance (*R*^2^ = 0.869–0.901), supporting the presence of energetically heterogeneous adsorption environments commonly associated with porous carbon materials containing structural defects, irregular pore structures, and oxygen-containing functional groups. Similar behavior has been reported for MB adsorption on heterogeneous biochar systems.^64^ The applicability of the Elovich model further suggests that adsorption-site energies are not uniformly distributed across the ACLDP surface, which is consistent with the heterogeneous adsorption behavior later observed from isotherm model discrimination.

In contrast, the PFO model exhibited relatively poor statistical agreement (*R*^2^ = 0.388–0.628), indicating that simple first-order adsorption kinetics cannot adequately represent the present adsorption system. Although the PSO model showed improved fitting performance compared with the PFO model (*R*^2^ = 0.724–0.791), it remained statistically less supported than the Weber–Morris and Avrami models, particularly according to AIC-based discrimination.

Importantly, the present results highlight the limitation of assigning adsorption mechanisms solely on the basis of traditional kinetic-model labels. While PSO behavior is frequently associated with surface-reaction-controlled adsorption, the weaker statistical support observed here suggests that adsorption kinetics in ACLDP cannot be interpreted exclusively through surface-interaction assumptions without simultaneously considering diffusion-assisted transport processes and mechanistic overlap among competing pathways.

From a statistical perspective, the lower AIC values observed for the Weber–Morris and Elovich models further confirm their stronger mechanistic support relative to the PFO and PSO models under the investigated conditions. More importantly, the present results demonstrate that multiple kinetic models may provide statistically acceptable fitting performance despite representing fundamentally different physical assumptions. Under such conditions, reliance solely on correlation coefficients may lead to oversimplified mechanistic interpretation in heterogeneous porous adsorption systems.

The Weber–Morris model is commonly used to evaluate diffusion-related transport behavior. Although the relatively high correlation coefficients suggest a major contribution from intraparticle diffusion, the fitted lines do not pass through the origin, indicating that intraparticle diffusion does not operate as the sole rate-limiting mechanism. Instead, the adsorption process likely proceeds through coupled and sequential stages involving external film diffusion, pore diffusion, progressive occupation of internal adsorption regions, and surface interactions acting simultaneously within the hierarchical pore network.^63^

The statistically preferred performance of diffusion-associated kinetic models is also physically consistent with the mesoporous structure identified from BET analysis, where mesopores likely function as transport-accessible pathways facilitating MB migration toward internal adsorption domains.

The kinetic parameters exhibit only minor variation across the investigated concentration range (58.51–117.01 mg L^−1^), suggesting that the overall adsorption mechanism remains consistent.

This variation remains within a relatively narrow range, indicating that no fundamental change in the controlling mechanism occurs over the investigated concentrations. However, a gradual decrease in adsorption rate at higher concentrations may be attributed to increased competition among MB molecules and partial saturation of available active sites. Overall, the adsorption kinetics of MB onto ACLDP can be interpreted as a coupled and multistage process governed by the interplay between diffusion-assisted transport, heterogeneous surface interactions, and progressive occupation of adsorption regions with different accessibility and energy distributions. The combined application of AIC and error-function analysis further demonstrates that adsorption behavior in heterogeneous biomass-derived porous carbons cannot be reliably interpreted using a single simplified kinetic assumption alone.

Importantly, the present framework does not treat adsorption models merely as empirical curve-fitting equations. Instead, the combined application of information-theoretic discrimination, error-function analysis, and physicochemical characterization enables mechanistically constrained interpretation of adsorption behavior within heterogeneous porous carbon systems. Under conditions where multiple models produce similarly high statistical agreement, AIC-based discrimination helps distinguish whether adsorption behavior is more consistent with homogeneous surface assumptions, heterogeneous energy distributions, diffusion-assisted transport, or multistage adsorption pathways. This integrated approach therefore provides mechanistic interpretability beyond conventional correlation-based fitting and reduces the risk of oversimplified adsorption assignments in structurally heterogeneous biomass-derived carbons.

Rather than merely identifying the mathematically best-fitting kinetic equation, the present statistical-mechanistic framework improves interpretation of the dominant transport pathways governing adsorption behavior within hierarchical porous carbon systems. These findings are also consistent with the structurally heterogeneous surface chemistry and hierarchical pore architecture identified through FTIR, Raman, BET, and electrokinetic analyses. The framework may also provide a more transferable basis for interpreting adsorption behavior across heterogeneous biomass-derived carbon systems where overlapping transport and surface-interaction mechanisms frequently complicate conventional model assignment.

### 3.5. Thermodynamics

#### 3.5.1. Activation energy

**Fig. 20:**
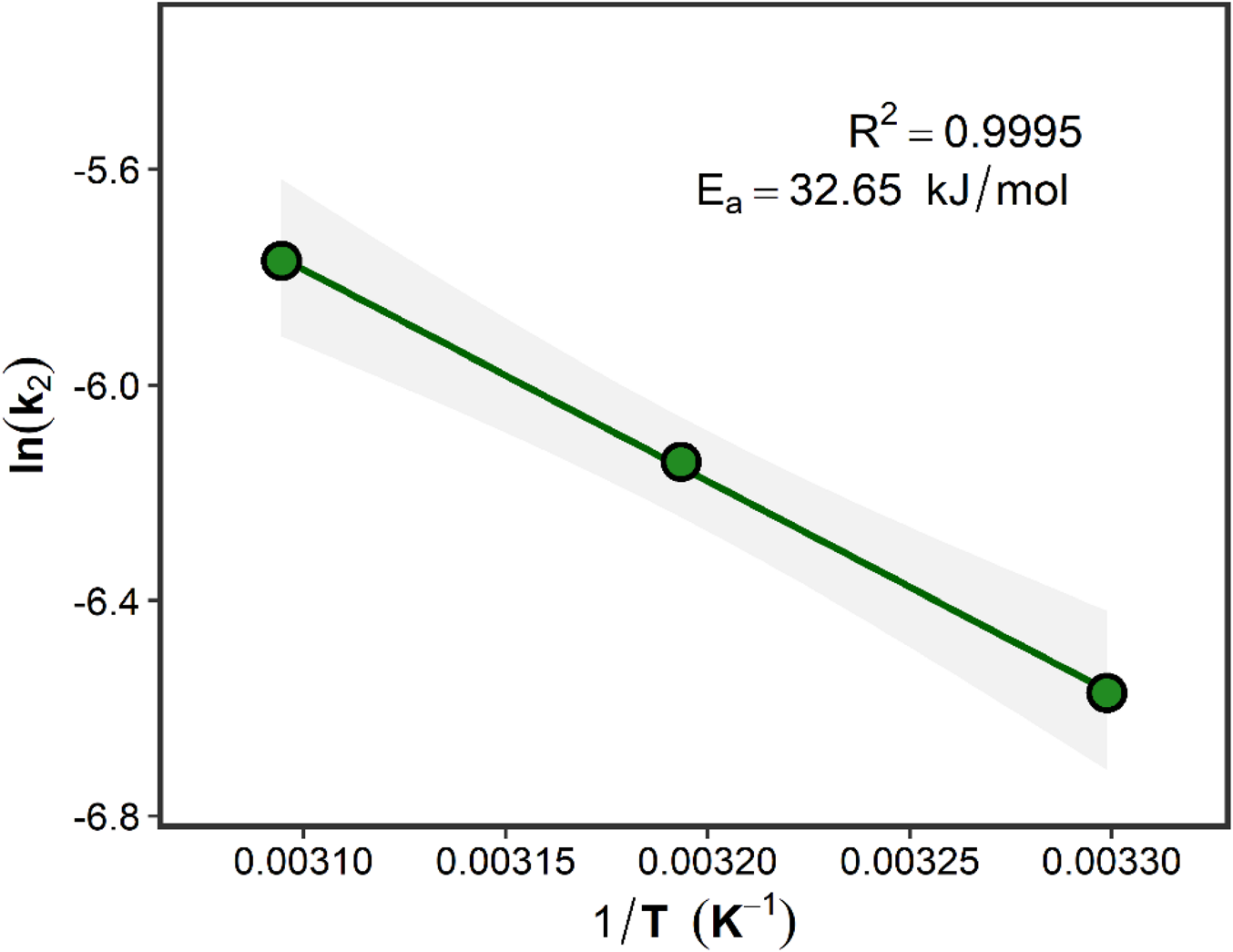
Plot showing the dependence of ln *k*_2_ on 1/*T*.

The activation energy (*E*_a_) for MB adsorption on ACLDP is presented in Fig. 20, with a value of approximately 32.65 kJ mol^−1^. This value provides information on the kinetic barrier associated with the adsorption process.

In adsorption systems, activation energy is often used to discuss the nature of the adsorption mechanism. Lower *E*_a_ values (typically below ∼40 kJ mol^−1^) are generally attributed to physisorption, whereas higher values may reflect stronger adsorbate–surface interactions. The obtained *E*_a_ value is consistent with adsorption systems governed by weak-to-moderate interaction energies. However, such classification should be interpreted with caution, as activation energy alone cannot fully distinguish between different adsorption mechanisms.

In combination with the kinetic analysis (Section 3.4), which indicates a multi-step process involving intraparticle diffusion and heterogeneous surface interactions, the present *E*_a_ value suggests that the adsorption process is not governed by a single mechanism, but rather by the combined effects of diffusion and surface interactions of varying strength. At the same time, the contribution of other interaction pathways cannot be excluded, particularly in the presence of surface functional groups on carbon materials.

This interpretation is consistent with previous studies on dye adsorption using porous carbon materials, where adsorption has been interpreted as involving coupled diffusion processes and relatively weak to moderate surface interactions.^65^

#### 3.5.2. Thermodynamic analysis of adsorption

**Fig. 21:**
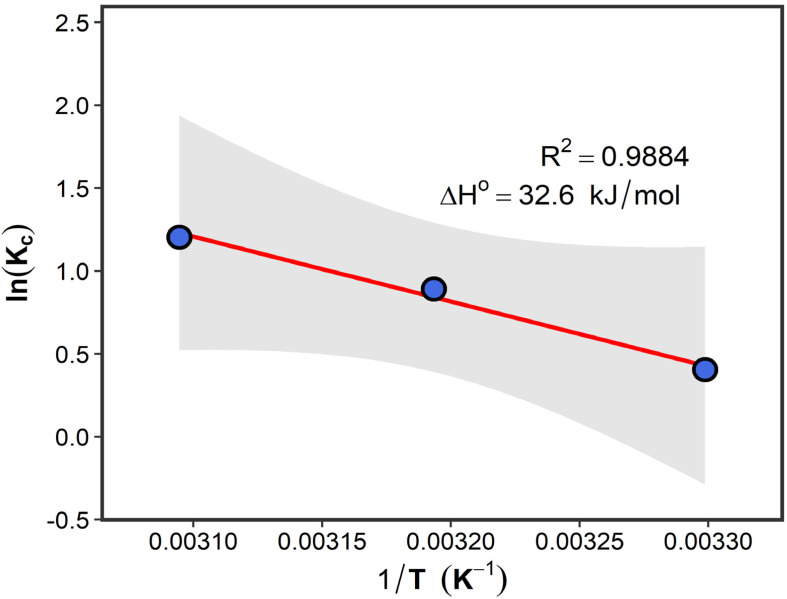
Plot of ln *K*_c_*versus* 1/*T*.

The thermodynamic parameters for MB adsorption on ACLDP are presented in Fig. 21 and Table 7.

**Table 7:** Thermodynamic parameters for MB adsorption on ACLDP

*T* (K)	Δ*G*° (kJ mol^−1^)	Δ*H*° (kJ mol^−1^)	Δ*S*° (kJ mol^−1^ K^−1^)
303	−1.02	32.60	0.1111
313	−2.32		
323	−3.23		

The negative values of Δ*G*° over the investigated temperature range suggest that the adsorption process is thermodynamically favorable. The magnitude of Δ*G*° becomes more negative with increasing temperature, indicating a temperature-dependent increase in adsorption favorability, which may be associated with enhanced mass transfer and diffusion at elevated temperatures. This behavior is consistent with the kinetic results (Section 3.4), where diffusion-related processes were found to play an important role in the overall adsorption mechanism. This behavior may arise from enhanced mass transfer at elevated temperatures, which can facilitate the movement of adsorbate molecules toward accessible sites within the pore structure. Similar observations have been reported for dye adsorption on porous carbon materials.^66^

The enthalpy change (Δ*H*° = 32.60 kJ mol^−1^) falls within the range typically observed for adsorption systems involving weak-to-moderate interactions. This value is consistent with adsorption systems in which multiple interaction mechanisms coexist, rather than being dominated by a single type of interaction. Comparisons with previous studies suggest that higher Δ*H*° values (*e.g.*, ∼68.7 kJ mol^−1^) generally reflect stronger adsorbate–surface interactions, whereas lower values (∼17–21 kJ mol^−1^) are typically associated with weaker interaction energies.^41,67^

The present value lies between these ranges, further supporting the interpretation that the adsorption process involves a combination of interaction types, consistent with the multi-step kinetic behavior discussed in Section 3.4.

In this context, interactions such as π–π interactions between aromatic structures, electrostatic effects, and hydrogen bonding may contribute to the adsorption process, although their relative contributions cannot be determined solely from thermodynamic data.^20^

The positive Δ*S*° value suggests an increase in disorder at the solid–liquid interface during adsorption. This behavior is often associated with the displacement of water molecules or hydrated ions from the adsorbent surface as MB molecules occupy adsorption sites, leading to an increase in the number of accessible microstates. Similar trends have been reported in aqueous dye adsorption systems.^68^

### 3.6. Proposed mechanisms

Based on the combined characterization results (FTIR, Raman spectroscopy, BET, and pH_pzc_), together with kinetic, isotherm, thermodynamic, and activation energy analyses, the adsorption of methylene blue (MB) onto ACLDP can be described as a synergistic, multi-stage process governed by coupled surface interactions and hierarchical transport phenomena, rather than a single dominant mechanism. This behavior reflects the interplay between surface heterogeneity and pore structure, which jointly control adsorption affinity and mass transfer efficiency.^49,50^

#### 3.6.1. Surface functional groups and electronic structure contributions

FTIR analysis indicates that oxygen-containing functional groups are involved in the adsorption process. The appearance of the band at 3119 cm^−1^ and the shift of the C=O band from 1703 to 1717 cm^−1^ suggest changes in the local chemical environment of surface groups after adsorption. Such spectral variations are commonly associated with interactions between adsorbates and oxygenated functional groups, including hydrogen bonding and dipole-related interactions.^27^ However, FTIR evidence remains indirect and does not allow unambiguous identification of specific interaction types. Therefore, these assignments should be interpreted as plausible rather than definitive, and considered in conjunction with other structural analyses.^27,29^

Raman spectroscopy provides complementary insight into the intrinsic carbon structure of ACLDP prior to adsorption. The presence of distinct D-band and G-band features, together with a broad 2D-band, indicates a defect-rich, partially graphitized carbon framework. The D-band reflects structural disorder and edge defects, while the G-band corresponds to sp^2^-hybridized graphitic domains. Such coexistence of defects and conjugated aromatic structures is characteristic of biomass-derived carbons and is known to generate a wide distribution of adsorption sites with different energy levels.^30,49^ While Raman analysis does not directly probe adsorbate–surface interactions, the presence of conjugated graphitic domains suggests that π–π electron donor–acceptor interactions between MB molecules and the carbon surface are structurally feasible and commonly reported for similar carbon-based adsorbents. Therefore, π–π interactions are considered a plausible contributing mechanism rather than being conclusively demonstrated by Raman evidence alone.^68^ Although the combined FT-IR, Raman, pH_pzc_, and zeta potential results provide indirect evidence supporting the possible involvement of electrostatic attraction, hydrogen bonding, π–π interactions, and pore-filling effects during methylene blue adsorption, these mechanisms should be interpreted cautiously because they were not independently verified using advanced surface-sensitive techniques such as XPS. Therefore, the proposed adsorption mechanism is intended as a plausible mechanistic interpretation rather than definitive proof of individual interaction pathways.

#### 3.6.2. Electrostatic interaction and initial adsorption stage

At pH 7, slightly above pH_pzc_ (6.3), the ACLDP surface carries a net negative charge due to partial deprotonation of –OH and –COOH groups. Under these conditions, electrostatic attraction between MB^+^ and negatively charged surface sites facilitates the initial adsorption step. Electrostatic attraction has been widely reported as a key driving force in the early stage of cationic dye adsorption on carbon materials.^69^ This interaction is likely to reduce the energy barrier for the approach of MB molecules to the surface, thereby contributing to faster adsorption kinetics at short contact times.^69,70^ Nevertheless, electrostatic interaction alone cannot fully explain the overall adsorption behavior, particularly under conditions of increased surface coverage or within internal pore regions.

#### 3.6.3. Hierarchical pore structure and diffusion-controlled transport

BET analysis shows that ACLDP possesses mesopores with an average size of ∼5.88 nm. These mesopores primarily function as transport pathways rather than dominant adsorption sites, facilitating molecular diffusion toward internal adsorption regions.^33^ The coexistence of mesopores and smaller pores suggests a hierarchical pore structure, which is critical for balancing adsorption capacity and mass transfer efficiency. This interpretation is supported by kinetic analysis, in which the Weber–Morris model provides the best overall fit, indicating that intraparticle diffusion plays a major, but not exclusive, role in controlling the adsorption rate. However, the deviation of fitted lines from the origin suggests that intraparticle diffusion is not the sole rate-limiting step, but rather operates in conjunction with other transport and surface processes.^63,66^

#### 3.6.4. Surface heterogeneity and kinetic behavior

Kinetic analysis shows that the adsorption process is not adequately described by pseudo-first-order or pseudo-second-order models. Instead, the Weber–Morris model exhibits the highest fitting performance, followed by the Avrami and Elovich models, based on combined statistical criteria (*R*^2^, RMSE, *χ*^2^, and AIC). The Elovich model provides moderate to good agreement (*R*^2^ = 0.869–0.901), indicating the presence of energetically heterogeneous adsorption sites.^63,64^ This is consistent with the structural characteristics of ACLDP, where FTIR and Raman analyses reveal the coexistence of functional groups, defects, and graphitic domains, leading to a broad distribution of adsorption energies.^30,49^ The Avrami model also shows strong agreement (*R*^2^ = 0.908–0.934), suggesting that the adsorption process involves multi-step pathways and complex adsorption kinetics, potentially arising from the interplay between surface reactions and diffusion processes.

#### 3.6.5. Isotherm behavior and energy distribution

Isotherm analysis shows that the Sips and Freundlich models provide the best fit (*R*^2^ ≈ 0.9975), indicating a heterogeneous surface with a non-uniform distribution of adsorption energies.^49,50^

#### 3.6.6. Thermodynamic and energetic considerations

Thermodynamic analysis shows that Δ*G*° < 0, confirming the spontaneous nature of the adsorption process. The Δ*H*° value (∼32.6 kJ mol^−1^) falls within an intermediate range, suggesting that adsorption involves a combination of physical interactions and weak chemical interactions, rather than purely physisorption.

This magnitude is consistent with adsorption systems governed predominantly by physisorption, with possible contributions from weak specific interactions such as electrostatic attraction and π–π interactions.

#### 3.6.7. Integrated adsorption mechanism

Taken together, the adsorption of MB onto ACLDP can be described as a coupled and hierarchical mechanism, in which:

(i) Electrostatic attraction facilitates the initial approach of MB molecules to the surface.^69,70^

(ii) Intraparticle diffusion is considered an important contributing process in the overall adsorption pathway, while mesoporous channels facilitate mass transfer toward internal adsorption regions.^63,66^

(iii) heterogeneous surface sites provide a distribution of adsorption energies.^49,50^

(iv) Multi-step adsorption pathways are involved, as supported by the applicability of the Avrami model.

(v) π–π interactions between MB and graphitic domains are considered structurally plausible but not directly confirmed.^68^

(vi) Hydrogen bonding and dipole interactions act as complementary secondary interactions.^27,29^

The relative contribution of these interactions should be interpreted as cooperative rather than individually dominant, and may vary depending on solution conditions and surface coverage. Unlike conventional adsorption studies that primarily identify the mathematically best-fitting model, the present work integrates information-theoretic model discrimination with multiscale structural and surface analyses to establish physically interpretable relationships between pore hierarchy, surface heterogeneity, diffusion-assisted transport, and adsorption energetics in biomass-derived porous carbons. The combined application of AIC, error-function analysis, nonlinear kinetic/isotherm modeling, and spectroscopic characterization demonstrates that MB adsorption onto ACLDP cannot be adequately described by a single simplified mechanism. Instead, adsorption proceeds through coupled pathways involving heterogeneous surface interactions, diffusion-assisted transport, pore accessibility effects, and structurally plausible π–π interactions within the hierarchical carbon framework. Importantly, several adsorption models produced similarly high *R*^2^ values despite representing fundamentally different physicochemical assumptions, highlighting the limitation of correlation-based interpretation alone. By simultaneously evaluating fitting quality and model complexity, the AIC framework enabled more reliable discrimination of adsorption pathways governed by heterogeneous surface energetics and multistage transport behavior.

Overall, the present statistical-mechanistic framework provides a more physically meaningful approach for interpreting competing adsorption and transport processes in heterogeneous biomass-derived porous carbon systems.

**Fig. 22:**
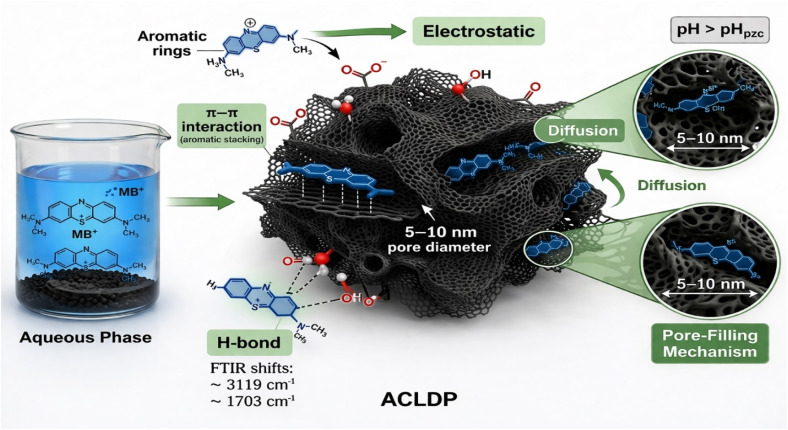
Mechanistic model of MB sequestration by ACLDP: integration of surface functional group interactions (π–π stacking, electrostatic interactions, and hydrogen bonding) and structural pore-related effects.

To provide a comprehensive visual representation, the proposed multi-mechanistic pathway for MB adsorption onto ACLDP, integrating surface chemistry and pore structure effects, is illustrated in Fig. 22.

### 3.7. Reusability & stability

**Fig. 23:**
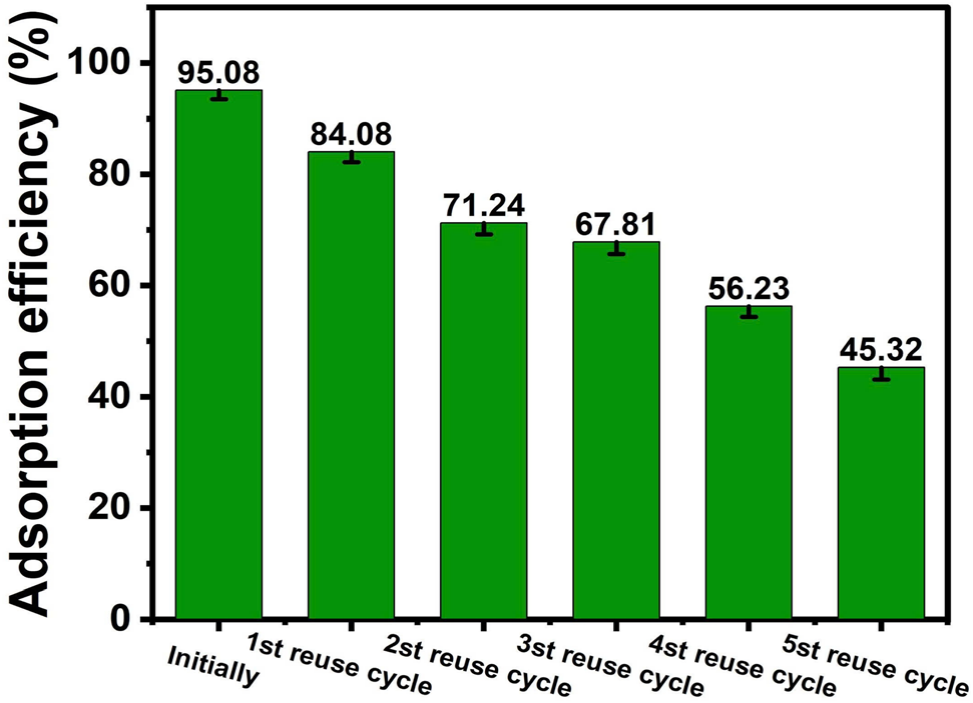
Reusability performance of ACLDP. Error bars represent standard deviation from triplicate experiments (*n* = 3).

The practical applicability of ACLDP was further examined through reusability tests and post-use structural analysis. The results of the reusability tests are shown in Fig. 23.

The adsorption efficiency of ACLDP decreases with successive reuse cycles, from 95.08% initially to 45.32% after five cycles. During the first reuse, removal remains relatively high (84.08%), indicating limited deterioration of accessible adsorption sites at this stage. From the second cycle onward, a progressive decline is observed (71.24% → 67.81% → 56.23% → 45.32%), suggesting gradual loss of adsorption performance. Several factors may account for this trend. Incomplete regeneration of active sites during desorption can lead to partial site blockage. Residual adsorbate species may remain attached, limiting site availability in subsequent cycles. Pore obstruction may also occur due to accumulation of retained molecules. This can reduce accessible surface area and hinder diffusion into internal regions. Repeated regeneration may additionally alter surface functional groups such as –OH and –COOH. These groups are often associated with interactions including hydrogen bonding, electrostatic attraction, and π–π interactions. Despite the observed decline, removal efficiency remains above 56.23% after four cycles. This suggests a moderate level of reusability under the tested conditions. Although ACLDP retained measurable adsorption performance after five regeneration cycles, partial reduction in removal efficiency was observed, potentially due to incomplete desorption and gradual alteration or blockage of accessible active sites during repeated adsorption–desorption cycles. XRD analysis after regeneration suggested that the primary carbon framework remained relatively stable. However, more comprehensive post-regeneration characterization, including SEM, FTIR, and BET analyses after multiple cycles, would be valuable for further evaluation of long-term structural stability and surface evolution. At the same time, the decreasing trend indicates limited long-term stability. The observed decline suggests that regeneration using ethanol alone may be insufficient to fully restore active sites, highlighting the need for improved regeneration strategies. Further improvement may require optimization of regeneration procedures. The choice of desorption solvent or the use of mild thermal treatment could help restore adsorption sites and reduce structural degradation.

#### 3.7.1. XRD analysis after reuse

**Fig. 24:**
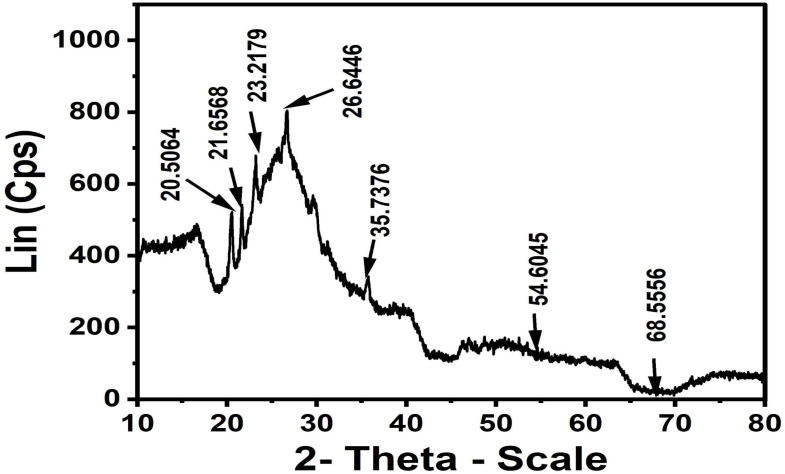
XRD pattern of ACLDP after two reuse cycles.

To further examine the structural origin of the observed performance decline, XRD analysis was conducted after repeated reuse cycles. The corresponding diffraction pattern is presented in Fig. 24. The XRD pattern of ACLDP after two reuse cycles retains features characteristic of amorphous carbon. Broad reflections appear at 2*θ* ≈ 20.51°, 21.66°, 23.22°, 26.64°, 35.74°, 54.60°, and 68.56°. Reflections in the range of 2*θ* ≈ 23–27°, particularly near 23.56° and 26.26°, are commonly associated with the (002) plane of disordered graphitic carbon, as reported for biochar materials.^18^ The features at 20.51° and 21.66° may relate to amorphous carbon domains or expanded interlayer spacing. Weak reflections at higher angles (35.74°, 54.60°, 68.56°) can be tentatively assigned to higher-order planes such as (100) or (101), reflecting limited structural ordering.^71^ After reuse, peak positions remain nearly unchanged. This suggests that the carbon framework is largely preserved. A reduction in peak intensity and slight peak broadening are observed. These changes may be associated with increased structural disorder or partial surface coverage by retained adsorbates.^72^ Such behavior points to reasonable structural stability, while also indicating a possible decrease in the number of accessible adsorption sites after repeated use.

### 3.8. Environmental implication

The conversion of *Lansium domesticum* peel into functional carbonaceous adsorbents exemplifies a waste-to-resource paradigm, offering a pathway to mitigate agricultural waste disposal challenges while potentially reducing reliance on conventional carbon sources. This strategy aligns with circular economy and sustainable material development principles by transforming low-value biomass into functional materials for environmental remediation. The synthesis route, integrating hydrothermal carbonization with H_3_PO_4_ activation, offers a potentially scalable framework, which may support future translation from bench-scale research to practical applications.

The adsorption performance observed for methylene blue (MB) underscores ACLDP's potential as a cost-effective candidate for treating dye-laden wastewater. In complex industrial matrices, such as textile effluents, the presence of competing ions and fluctuating pH conditions generally requires an adsorbent with both structural accessibility and chemical resilience. In this regard, the hierarchical mesopore network (∼5.88 nm) of ACLDP may facilitate efficient intraparticle diffusion, while its oxygen-rich surface functionalities contribute to adsorption affinity toward cationic pollutants. This cooperative mechanism may contribute to maintaining adsorption performance in dynamic aqueous environments. While the present work demonstrates the adsorption capability of ACLDP, future investigations should prioritize regeneration cycles and continuous-flow column studies to fully validate its long-term stability and practical feasibility in large-scale water treatment systems.

### 3.9. Limitations and future perspectives

To ensure a balanced interpretation of the present findings, several limitations should be considered. First, the adsorption experiments were conducted in single-solute systems, which may not fully represent the complexity of real-world wastewater containing competing ions, natural organic matter, and variable pH conditions. These factors may significantly influence adsorption behavior and consequently affect both efficiency and selectivity under practical conditions. The present work focuses specifically on methylene blue as a representative cationic dye. Although MB is widely employed as a benchmark adsorbate for porous carbon materials, the adsorption behavior toward other cationic or anionic dyes may differ due to variations in molecular size, charge distribution, and functional groups. Future studies should therefore evaluate adsorption selectivity and applicability using structurally diverse dye systems under multi-component conditions. Second, the observed decline in adsorption performance upon reuse suggests incomplete regeneration and possible alterations in surface properties or pore accessibility, indicating that regeneration protocols require further optimization to ensure long-term stability.

Third, the proposed adsorption mechanism is largely inferred from indirect evidence derived from spectroscopic and physicochemical analyses. Advanced surface-sensitive techniques, such as X-ray photoelectron spectroscopy (XPS) or *in situ* analytical methods, were not employed in this study, which may limit detailed insight into surface-level interactions.

Future research should therefore focus on evaluating adsorption performance in multi-component systems and under realistic operating conditions. In addition, the development of continuous-flow processes and improved regeneration strategies will be important for practical implementation. Extending the application of ACLDP to other classes of emerging pollutants, such as antibiotics and heavy metals, would further elucidate its structural versatility and expand its environmental applicability.

## 4. Conclusion

This study reports the successful synthesis of a hierarchically porous activated carbon (ACLDP) from *Lansium domesticum* peel *via* a combined hydrothermal and H_3_PO_4_ activation approach, resulting in a mesopore-rich carbon framework with abundant surface functional groups. The prepared material exhibits a high adsorption capacity for methylene blue (*q*_max_ ≈ 345.8 mg g^−1^) despite its relatively moderate surface area, indicating that adsorption performance is not solely determined by surface area but is also influenced by pore architecture and surface chemistry.

The integration of nonlinear modeling with Akaike Information Criterion (AIC)-based statistical selection provides a robust and quantitative framework for adsorption analysis, enabling more reliable model discrimination beyond conventional goodness-of-fit metrics. The results suggest that adsorption behavior can be described by the combined contributions of heterogeneous surface interactions and diffusion-related transport processes, rather than a single dominant mechanism. Instead, a multi-step and condition-dependent adsorption behavior is observed, arising from the interplay between mesopore-facilitated diffusion and surface functional group-mediated interactions.

Thermodynamic and activation energy analyses further suggest that weak to moderate interactions play a significant role in the adsorption process, supporting the coexistence of physical and chemical contributions. These findings indicate that rational tuning of pore hierarchy and surface functionality can help mitigate limitations associated with relatively low surface area, providing useful insights for adsorbent design.

Overall, this work suggests a promising pathway for valorizing agricultural waste into carbon-based adsorbents and provides a statistically grounded framework for adsorption analysis. The results offer practical guidance and potentially transferable insights for the rational design of next-generation adsorbents for wastewater treatment within a circular economy framework.

## Author contributions

Tra Huong Do engaged in the conceptualization, methodology, and manuscript preparation. Truong Xuan Vuong contributed significantly to data interpretation and writing and editing manuscript. Nguyen Ngoc Phuong Ngan, Mai Xuan Truong, Tran Thi Hue. Do Quoc Dung contributed equally to data collection, analysis, and manuscript review. All authors approved the final version of the manuscript.

## Conflicts of interest

The authors declare no conflicts of interest.

## Supplementary Material

RA-016-D6RA02695H-s001

## Data Availability

The data supporting this article are provided in the supplementary information (SI). Additional information is available from the corresponding author upon reasonable request. Supplementary information: the calibration curve for methylene blue (MB) determination (Fig. S1), along with the effects of pH (Fig. S2), contact time at different initial concentrations (Fig. S3), adsorbent dosage (Fig. S4), temperature at 303–323 K (Fig. S5), and initial MB concentration (Fig. S6) on the adsorption performance of the prepared adsorbent. See DOI: https://doi.org/10.1039/d6ra02695h.
