# Supplementary material for: Hierarchically porous activated carbon derived from Lansium domesticum peel via hydrothermal-H3PO4 activation for enhanced methylene blue removal: adsorption behavior, advanced modeling and mechanistic insights
Source: RSC Adv. 2026 May 22;16(30):28004–35. doi: 10.1039/d6ra02695h (PMC13202429; doi:10.1039/d6ra02695h)
Supplement: RA-016-D6RA02695H-s001 [file RA-016-D6RA02695H-s001.pdf]

Supplementary Information (SI) for RSC Advances.
This journal is © The Royal Society of Chemistry 2026

Supplementary Information

Title: Hierarchically porous activated carbon derived from *Lansium domesticum* peel via hydrothermal–H_3PO_4 activation for enhanced methylene blue removal: adsorption behavior, advanced modeling and mechanistic insights

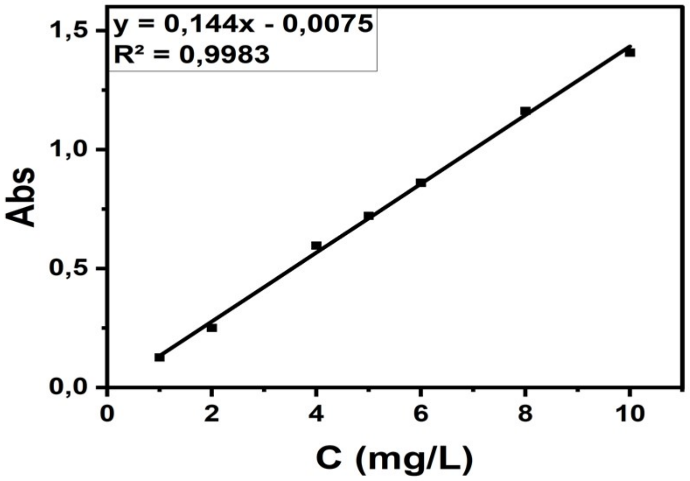

Figure S1. Calibration curve for the determination of methylene blue (MB) concentration.

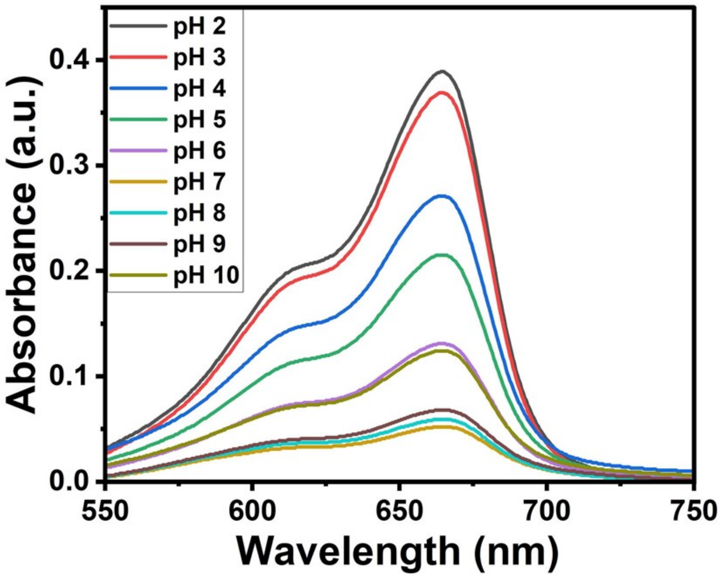

Figure S2. Effect of pH on the adsorption capacity.

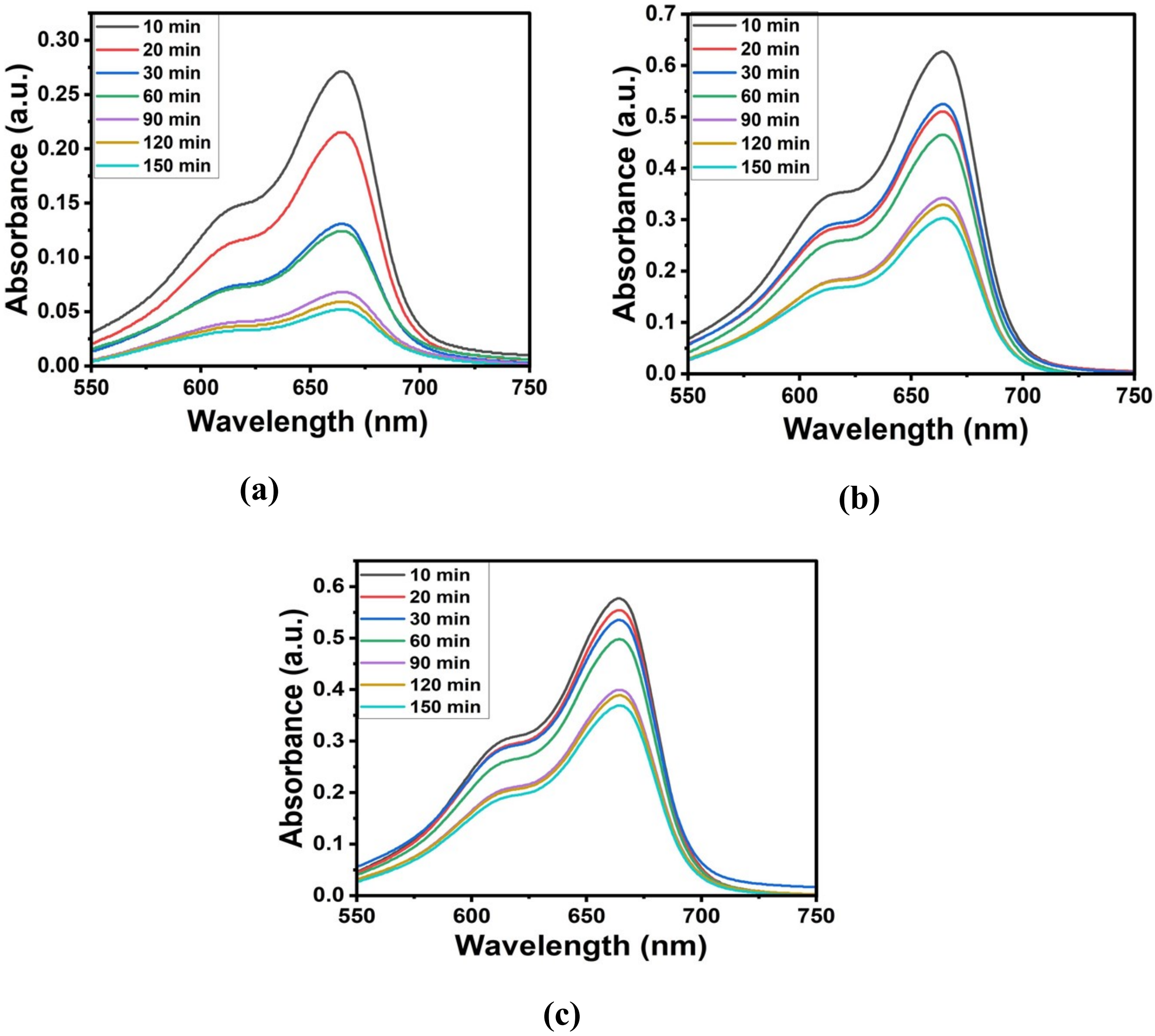

Figure S3. Effect of contact time on adsorption capacity at initial concentrations of 58.51 mg/L (a), 87.76 mg/L (b), and 117.01 mg/L (c).

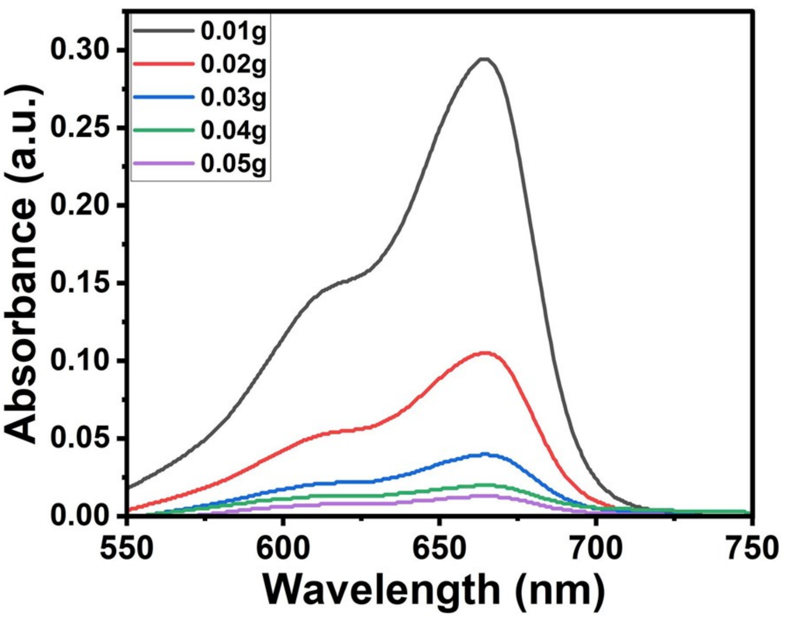

Figure S4. Effect of adsorbent dosage on adsorption capacity.

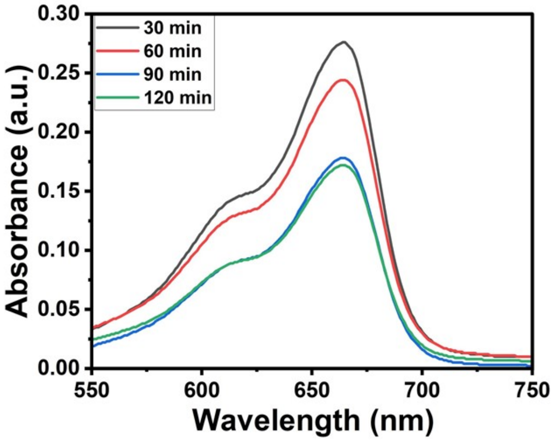

(a)

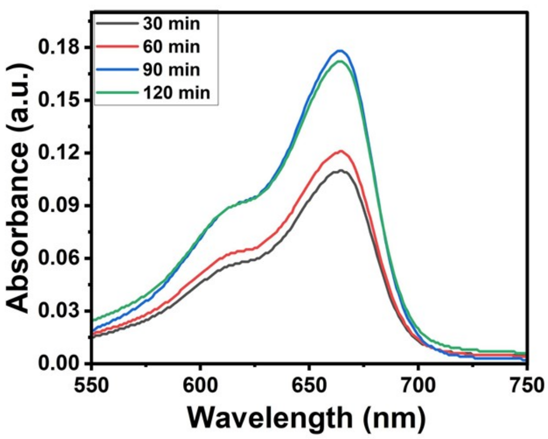

(b)

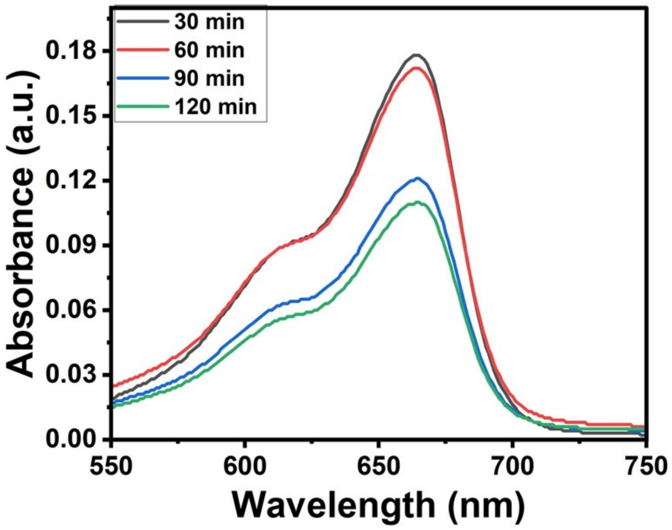

(c)

Figure S5. Effect of temperature on adsorption capacity at 303 K (a), 313 K (b), and 323 K (c).

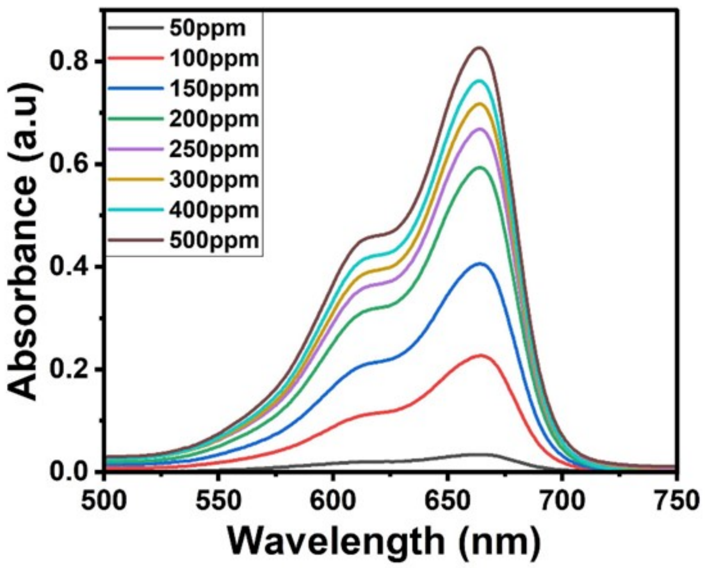

Figure S6. Effect of initial concentration on adsorption capacity.
